# Epidemiological Analysis and 2040 Projections of Atrial Fibrillation and Flutter in Asia

**DOI:** 10.1016/j.jacasi.2026.06.037

**Published:** 2026-08-20

**Authors:** Jing Chen, Shunyu Yao, Zhongshan Xiong, Linfeng Xie, Yuanzhu Li, Gregory Y.H. Lip, Bi Huang

**Affiliations:** a Department of Cardiovascular Medicine, Cardiovascular Research Center, The First Affiliated Hospital of Chongqing Medical University, Chongqing, China; b Cardiovascular Disease Laboratory, Chongqing Medical University, Chongqing, China; c Liverpool Centre for Cardiovascular Science at University of Liverpool, Liverpool John Moores University, Liverpool Heart & Chest Hospital, Liverpool, United Kingdom; d Department of Clinical Medicine, Aalborg University, Aalborg, Denmark; e Medical University of Bialystok, Bialystok, Poland

**Keywords:** atrial fibrillation and flutter (AF/AFL), Asia, epidemiological analysis, projections

## Abstract

**Background:**

Atrial fibrillation and flutter (AF/AFL) impose a substantial and growing global burden, yet comprehensive assessments focusing on Asia, accounting for regional heterogeneity, demographic drivers, and future trajectories, remain limited.

**Objectives:**

This study aims to systematically evaluate the burden of AF/AFL across Asia from 1990 to 2021, to quantify the contributions of demographic and epidemiological drivers, and to project future trends to inform region-specific prevention strategies.

**Methods:**

Using Global Burden of Disease 2021 data, we analyzed prevalence, incidence, mortality, and disability-adjusted life years (DALYs) of AF/AFL across 48 Asian countries and territories. Joinpoint regression assessed temporal trends; decomposition analysis quantified contributions of population growth, aging, and epidemiological changes; and auto-regressive integrated moving average models projected burden to 2040.

**Results:**

In 2021, Asia had 25,739,093 (95% uncertainty interval [UI]: 20,431,798-33,586,165) prevalent cases of AF/AFL, with age-standardized rate 529.04 (95% UI: 420.26-680.91) per 100,000. Southeast Asia exhibited the highest age-standardized burden, while only the high-income Asia-Pacific region showed declining trends. Although the burden on male was greater, older females were a group that could not be overlooked. Joinpoint analysis revealed a nonlinear trajectory with accelerated growth after 2014. Decomposition analysis identified population growth and aging as primary drivers. High systolic blood pressure was the leading risk factor, with high body mass index showing the most rapid increase. Projections indicate sustained rises through 2040, with particularly pronounced increases among females.

**Conclusions:**

AF/AFL has become an increasingly severe public health challenge in Asia. Therefore, tailored prevention and control strategies should be developed according to regional characteristics to effectively alleviate its disease burden.

Atrial fibrillation and flutter (AF/AFL) is the most common heart rhythm disorder and is associated with mortality and morbidity from stroke, dementia, heart failure, and death, as well as hospitalizations.^1^^,^^2^ The Global Burden of Disease (GBD) 2021 has indicated that the estimated number of cases of AF/AFL worldwide is 52,552,045, with 4,484,926 new cases and 338,947 deaths.^3^ Between 1990 and 2021, the global prevalence of AF/AFL increased by 137%.^4^

Epidemiological studies have indicated that AF/AFL has a racial disparity, with individuals of European descent demonstrating a higher incidence compared to those of Asian or African ancestry.^5^ Asian individuals exhibit lower average body weight and differences in the pharmacogenomic characteristics of key drug-metabolizing enzymes, such as CYP2C9 and VKORC1.^6^^,^^7^ As regards complications, racial differences also in stroke and bleeding have been reported in ecological epidemiological studies.^8^^,^^9^ As the most densely populated region in the world, Asia exhibits unique epidemiological characteristics with regard to AF/AFL. Previous studies have found that East Asian populations appear to have a lower propensity to thrombosis but a higher risk of bleeding, alongside a higher prevalence of complications such as hypertensive heart disease and hemorrhagic stroke.^10^^,^^11^ Additionally, substantial heterogeneity in economic development across various regions in Asian nations has led to pronounced disparities in health care delivery systems, policy support, and resource allocation.^12^ These factors contribute to varying levels of health awareness among the population, the diagnosis and treatment access of AF/AFL, and the differences in prognosis and health outcomes within this region.

Although global burden studies have updated the epidemiology of AF/AFL and highlighted health inequalities globally, they did not specifically examine the heterogeneous patterns across Asian subregions or quantify the relative contributions of demographic and epidemiological drivers within this continent.^3^ A recent Asia-Pacific review underscored the high absolute number of AF cases but relied on heterogeneous national estimates without standardized longitudinal analyses or future projections.^13^ Country-specific investigations in China offered valuable insights yet were confined to a single nation.^14^^,^^15^ Moreover, while metabolic risk factors have been identified as key global contributors, their Asia-specific stratification remains unaddressed.^16^ To address these gaps, we therefore used the GBD 2021 database (https://ghdx.healthdata.org/gbd-2021) to conduct a comprehensive assessment of the burden of AF/AFL across 48 Asian countries and regions between 1990 and 2021, with a view to developing a series of targeted prevention strategies. Ethical approval was waived for this study, as it used anonymized, publicly available data.

## Methods

### Data source

This study draws on data from the GBD 2021, which provides comprehensive estimates for 371 diseases and injuries and 88 risk factors across 204 countries and territories.^17^ Data inputs included vital registration systems, disease registries, hospital records, surveys, and published studies, which were processed using standardized modeling tools.^17^ AF/AFL was defined using the International Classification of Diseases (ICD) code (ICD-10: I48-I48.92). We employed on the incidence, prevalence, mortality, and disability-adjusted life years (DALYs) for AF/AFL from 1990 to 2021 across 48 Asian countries and territories, disaggregated by sex and age. These data are publicly available through the Global Health Data Exchange (GHDx) query tool.^18^

### Age standardization

To eliminate the influence of age structure and improve data comparability, we calculated age-standardized rates (ASRs) for prevalence (ASPR), incidence (ASIR), mortality (ASMR), and DALYs using the direct method. The formula is as follows^17^:

$$ASR=\frac{\sum_{i=1}^{A}a_{i}w_{i}}{\sum_{i=1}^{A}w_{i}}\times 1000,000$$

where: *a*_*i*_ is number of events in the *i*th age group; *w*_*i*_ is weight of the *i*th age group in the GBD standard population; *A* is total number of age groups.

### Disability-adjusted life years

DALYs represent the sum of years of life lost because of premature mortality and years lived with disability, providing a composite measure that quantifies the impact of both fatal and nonfatal health outcomes.^17^

### Risk factors of AF/AFL

We quantified the impact of environmental/occupational, behavioral, and metabolic risk factors on the burden of AF/AFL in Asia. Following the GBD comparative risk assessment framework, we extracted all third-level risk factors. Based on established evidence of causal relationships, the GBD defines 6 risk factors as contributors to AF/AFL burden: high body mass index (BMI), high systolic blood pressure (SBP), high alcohol use, dietary risks (diet high in sodium), tobacco use (smoking), and other environmental risks. To validate our selection, we reviewed the literature and confirmed that each of these risk factors has been independently associated with AF/AFL in prior epidemiological studies.^19^, ^20^, ^21^, ^22^, ^23^ These risk factor–outcome pairs were therefore included in our final analysis to evaluate their attributable mortality and DALYs in Asian populations with AF/AFL.

### Statistical analysis

To address issues of heterogeneity in diagnostic capacity, death registration systems, and cause-of-death attribution, particularly the high proportion of “garbage coding” deaths in areas with weak data infrastructure, the GBD study employed a series of robust methodological approaches.^24^ Through systematic data extraction and collation, the study identified and harmonized input data sources; in areas with sparse or missing data, spatio-temporal Gaussian process regression was used to smooth the estimates.^25^ Core epidemiological parameters, including incidence, prevalence, mortality, and DALYs, were modeled using the DisMod-MR 2.1 Bayesian meta-regression framework. The CODEm model was employed for estimating cause-specific mortality rates, along with an algorithm for the systematic reallocation of miscoded data.^26^^,^^27^ This model assumes a negative binomial likelihood to account for overdispersion and employs Markov chain Monte Carlo for estimation, while standard convergence diagnostics are used to assess model fit. Further details regarding data processing methods can be found in previous studies.^24^

In terms of quantifying uncertainty, the GBD study generated 1,000 samples for each parameter to capture both sampling and nonsampling errors simultaneously, and assessed the reliability of the estimates on this basis.^27^ Furthermore, the study introduced a 95% uncertainty interval (UI), employing Bayesian methods to repeatedly sample the distributions of input data and model parameters, generating 1,000 plausible values for each outcome metric, with the 2.5th and 97.5th percentiles serving as the lower and upper bounds of the interval.^17^ This interval indicates that, given the model and existing data, there is a 95% probability that the true value lies within this range.

### Descriptive analysis

Five major subregions of Asia and 48 countries and territories (listed in Supplemental Table 1) were included to assess geographical variation. Temporal trends from 1990 to 2021 were evaluated to describe the evolution of disease burden. Stratified analyses by sex and age were conducted to characterize demographic disparities. Since the onset age for AF/AFL in Asian populations is 30 years according to the database, we divided the population into 14 age subgroups: 30-34, 35-39, 40-44, …, 95+ years.

### Joinpoint regression analysis

To estimate the temporal trends of AF/AFL, we employed a segmented linear regression approach. Specifically, using the *segmented* package in R, a model was constructed with the log-transformed ASR as the dependent variable and year as the independent variable; the time points at which significant changes in trend occurred were identified using an automatic break-point detection procedure.^28^ Following model fitting, the slopes of each segment were exponentially transformed to yield the annual percentage change (APC); furthermore, the APCs were weighted by the length of each segment and summed to obtain the average annual percentage change (AAPC).^29^ This analysis was performed using R version 4.4.2. A trend was classified as increasing if the lower limit of the 95% confidence interval for AAPC was greater than zero, decreasing if the upper limit was less than zero, and stable if the confidence interval included zero.

### Decomposition analysis

We employed the Das Gupta decomposition method to systematically quantify the key drivers of changes in AF/AFL burden across Asian subregions between 1990 and 2021. This technique is a deterministic mathematical attribution approach with the inherent property of perfect additivity. Consistent with its standard analytical framework, interaction terms arising from concurrent changes across multiple drivers are proportionally allocated to 3 core components, including population growth, aging, and epidemiological change, with no separate residual term retained. By design, the sum of the 3 components is strictly equivalent to the total net change in disease burden, accounting for 100% of the observed variation. This decomposition enables estimation of the individual contribution of each factor to the overall burden change, thereby elucidating the drivers underlying heterogeneous AF/AFL burden trajectories across subregions. Given that the method performs a mathematical partition of empirical data rather than statistical inference of regression parameters, significance testing for individual components is not applicable.

### ARIMA forecasting

To project future trends in AF/AFL burden in Asia, we employed the auto-regressive integrated moving average (ARIMA) model based on historical data from 1990 to 2021.^30^^,^^31^ This method integrates 3 components: the auto-regressive (AR) component captures the sustained influence of historical disease burden; the integrated (I) component uses differencing to remove long-term trends and seasonal effects; and the moving average (MA) component captures the linear relationship between current observations and past forecast errors.^32^^,^^33^ The ARIMA framework is well-suited for medium- to long-term forecasting of time-series data.^30^ The resulting projections provide critical evidence to inform public health planning and guide resource allocation strategies across Asian subregions.^31^

All statistical analyses were performed using R version 4.4.2. A 2-sided *P* value of <0.05 was considered statistically significant.

## Results

### AF/AFL burden in Asia and subregions

In 2021, Asia had an estimated 25,739,093 AF/AFL cases (95% UI: 20,431,798-33,586,165), corresponding to an ASPR of 529.04 per 100,000 population (95% UI: 420.26-680.91). The prevalence showed an increasing trend, with a percentage change (PC) of 0.04 (95% UI: 0.02-0.06) between 1990 and 2021. There were 2,316,760 incident cases (95% UI: 1,795,505-3,052,653) and an ASIR of 46.69 per 100,000 (95% UI: 35.91-61.67). Incidence remained stable overall, with a PC of 0.01 (95% UI: −0.01 to 0.03). A total of 153,470 deaths (95% UI: 127,841-172,464) were recorded, yielding an ASMR of 3.84 per 100,000 (95% UI: 3.16-4.33). Mortality also exhibited a stable trend, with a PC of 0.05 (95% UI: −0.14 to 0.28). The disease burden of DALYs was 4,026,948 (95% UI: 3,269,416-4,982,293), with an age-standardized DALYs rate of 87.96 per 100,000 (95% UI: 72.78-107.27). The PC for DALYs was 0.03 (95% UI: −0.08 to 0.15), indicating overall stability (Table 1).

**Table 1:** Prevalence, Incidence, Mortality, and DALYs of AF/AFL in Asia

	Counts (95% UI, 1990)	ASR per 100,000 (95% UI, 1990)	Counts (95% UI, 2021)	ASR per 100,000 (95% UI, 2021)	PC (95% UI, 1990-2021)	AAPC (95% CI)
Prevalence						
Asia	8,735,972 (6,929,130-11,403,331)	507.59 (401.04-652.62)	25,739,093 (20,431,798-33,586,165)	529.04 (420.26-680.91)	0.04 (0.02-0.06)	0.159^a^ (0.124-0.194)
Male	4,606,100 (3,639,372-5,969,649)	562.48 (445.88-722.46)	13,401,943 (10,638,584-17,397,194)	589.46 (467.45-762.42)	0.05 (0.02-0.08)	0.187^a^ (0.154-0.220)
Female	4,129,872 (3,266,793-5,407,883)	459.53 (360.25-593.62)	12,337,150 (9,769,353-15,906,473)	475.28 (374.54-611.44)	0.03 (0.02-0.05)	0.106^a^ (0.066-0.146)
Central Asia	222,460 (171,711-290,357)	513.48 (395.59-666.95)	392,829 (305,783-509,896)	536.72 (410.76-698.74)	0.05 (0.02-0.07)	0.150^a^ (0.142-0.159)
East Asia	3,359,584 (2,649,140-4,376,801)	462.71 (363.30-601.04)	11,215,165 (8,885,909-14,572,495)	526.44 (420.60-683.56)	0.14 (0.10-0.18)	0.434^a^ (0.354-0.514)
High-income Asia Pacific	1,052,102 (825,876-1,364,552)	533.61 (423.32-688.79)	2,165,450 (1,737,905-2,727,227)	465.22 (383.84-577.04)	−0.13 (−0.17 to −0.07)	−0.288^a^ (−0.382 to −0.194)
South Asia	2,357,498 (1,827,475-3,099,820)	519.49 (401.88-683.87)	6,882,583 (5,295,961-9,026,139)	530.90 (410.19-696.69)	0.02 (0.01-0.03)	0.078^a^ (0.060-0.096)
SE Asia	1,377,746 (1,089,207-1,799,890)	637.21 (502.03-823.86)	3,898,608 (3,091,004-5,118,881)	662.33 (519.61-854.36)	0.04 (0.03-0.05)	0.127^a^ (0.121-0.133)
Incidence						
Asia	847,177 (658,303-1,110,926)	46.28 (35.31-61.36)	2,316,760 (1,795,505-3,052,653)	46.69 (35.91-61.67)	0.01 (−0.01 to 0.03)	0.067^a^ (0.033-0.100)
Male	437,534 (340,206-566,024)	48.42 (37.11-63.93)	1,160,175 (897,489-1,527,484)	48.66 (37.83-63.90)	0.01 (−0.02 to 0.03)	0.052^a^ (0.013-0.091)
Female	409,643 (314,735-541,435)	43.82 (33.02-58.29)	1,156,585 (885,253-1,540,837)	44.32 (33.87-59.02)	0.01 (−0.01 to 0.03)	0.045^a^ (0.006-0.084)
Central Asia	20,049 (15,184-26,387)	44.05 (33.40-58.03)	36,231 (27,486-47,288)	44.53 (33.54-57.85)	0.01 (−0.01 to 0.04)	0.043^a^ (0.032-0.055)
East Asia	321,766 (246,730-424,271)	42.98 (32.71-56.93)	953,898 (737,314-1,249,815)	45.11 (35.12-59.61)	0.05 (0.01-0.09)	0.179^a^ (0.108-0.249)
High-income Asia Pacific	86,518 (66,803-113,257)	43.22 (33.54-56.82)	152,471 (116,375-199,323)	36.27 (29.16-46.41)	−0.16 (−0.20 to −0.11)	−0.409^a^ (−0.487 to −0.330)
South Asia	249,150 (190,686-331,288)	50.08 (37.35-66.50)	698,920 (527,866-934,818)	51.01 (38.09-67.89)	0.02 (0.01-0.03)	0.068^a^ (0.050-0.086)
SE Asia	135,279 (105,015-176,227)	58.77 (44.77-78.09)	369,518 (285,921-483,602)	59.66 (45.60-79.28)	0.02 (0.00-0.03)	0.053^a^ (0.046-0.059)
Deaths						
Asia	39,389 (32,588-48,147)	3.64 (3.02-4.47)	153,470 (127,841-172,464)	3.84 (3.16-4.33)	0.05 (−0.14 to 0.28)	0.173^a^ (0.062-0.283)
Male	15,598 (11,941-18,762)	3.28 (2.55-3.97)	61,178 (53,814-69,904)	3.78 (3.29-4.32)	0.15 (−0.07 to 0.52)	0.401^a^ (0.279-0.523)
Female	23,791 (19,544-30,412)	3.87 (3.16-4.93)	92,292 (73,137-107,767)	3.86 (3.05-4.51)	0.00 (−0.21 to 0.26)	0.046 (−0.080 to 0.172)
Central Asia	684 (596-799)	1.87 (1.61-2.20)	1,386 (1,241-1,519)	2.37 (2.09-2.60)	0.27 (0.07-0.47)	1.026^a^ (0.577-1.474)
East Asia	17,272 (14,013-21,292)	4.94 (3.92-6.07)	67,666 (54,232-80,814)	4.30 (3.42-5.18)	−0.13 (−0.34 to 0.13)	−0.404^a^ (−0.628 to −0.179)
High-income Asia Pacific	5,018 (4,465-5,373)	3.06 (2.68-3.30)	17,112 (13,415-19,331)	2.46 (2.01-2.74)	−0.20 (−0.28 to −0.14)	−0.748^a^ (−0.901 to −0.595)
South Asia	7,594 (4,655-11,542)	2.38 (1.46-3.62)	36,165 (27,041-46,073)	3.69 (2.75-4.71)	0.55 (0.15-1.11)	1.592^a^ (1.324-1.860)
SE Asia	6,120 (5,116-7,566)	4.24 (3.50-5.30)	22,401 (18,974-25,759)	5.27 (4.39-6.12)	0.24 (−0.02 to 0.54)	0.672^a^ (0.611-0.733)
DALYs						
Asia	1,296,203 (1,017,915-1,633,974)	85.07 (68.74-106.21)	4,026,948 (3,269,416-4,982,293)	87.96 (72.78-107.27)	0.03 (−0.08 to 0.15)	0.109^a^ (0.065-0.153)
Male	626,317 (473,170-800,311)	85.91 (66.63-107.12)	1,929,999 (1,539,206-2,399,928)	93.05 (76.06-113.59)	0.08 (−0.04 to 0.25)	0.265^a^ (0.206-0.323)
Female	669,887 (535,453-844,668)	83.38 (67.73-104.17)	2,096,949 (1,675,414-2,577,093)	83.12 (66.48-101.85)	0.00 (−0.13 to 0.14)	−0.005 (−0.074 to 0.063)
Central Asia	27,964 (21,497-36,702)	66.28 (51.57-86.71)	52,600 (41,314-67,337)	75.00 (59.90-95.50)	0.13 (0.06-0.22)	0.398^a^ (0.212-0.584)
East Asia	533,837 (416,978-668,275)	93.83 (76.48-115.88)	1,723,468 (1,364,785-2,143,575)	89.83 (72.42-109.59)	−0.04 (−0.19 to 0.13)	−0.092 (−0.213 to 0.028)
High-income Asia Pacific	156,515 (125,660-193,831)	83.08 (67.38-102.03)	364,512 (301,067-443,767)	69.03 (56.40-84.74)	−0.17 (−0.22 to −0.13)	−0.504^a^ (−0.652 to −0.357)
South Asia	311,117 (228,511-416,726)	71.78 (52.92-96.69)	1,071,260 (820,127-1,366,680)	88.30 (68.02-111.45)	0.23 (0.06-0.43)	0.725^a^ (0.602-0.849)
SE Asia	200,296 (158,700-252,961)	102.35 (82.98-128.37)	612,969 (501,403-745,497)	115.40 (95.88-139.11)	0.13 (−0.01 to 0.27)	0.368^a^ (0.333-0.402)

The table presents the trends from 1990 to 2021 in prevalence, incidence, mortality and DALYs for AF/AFL across Asia in by sex and subregion, along with corresponding AAPC values derived from joinpoint regression analysis. With the exception of the high-income Asia Pacific region, which has experienced a decline in disease burden, the burden has remained stable or increased across all other regions, with Southeast Asia carrying the greatest burden. All data were obtained from the open database of the Global Burden of Disease study in the Global Health Data Exchange. Percentage change is the difference between the 2021 and 1990 values, divided by the 1990 value.

AAPC = annual average percentage change; AF/AFL = atrial fibrillation and flutter; ASR = age-standardized rate; DALYs = disability-adjusted life years; PC = percentage change; SE Asia = Southeast Asia; UI = uncertainty interval.

a Results are statistically significant (*P* < 0.001).

Regional disparities in the burden of AF/AFL were observed across Asia in 2021. East Asia reported the highest prevalent cases (11,215,165; 95% UI: 8,885,909-14,572,495), incident cases (953,898; 95% UI: 737,314-1,249,815), deaths (67,666; 95% UI: 54,232-80,814), and DALYs cases (1,723,468; 95% UI: 1,364,785-2,143,575). However, after ASR, Southeast Asia exhibited the highest disease burden, with an ASPR of 662.33 per 100,000 (95% UI: 519.61-854.36), an ASIR of 59.66 (95% UI: 45.60-79.28), an ASMR of 5.27 (95% UI: 4.39-6.12), and an age-standardized DALYs rate of 115.40 (95% UI: 95.88-139.11) (Table 1). Trend analyses from 1990 to 2021 revealed significant heterogeneity across Asian regions. Most regions experienced stable or increasing trends in AF/AFL, excepting the high-income Asia Pacific region, which showed declining trends (all PC < 0) (Table 1, Supplemental Figure 1).

### AF/AFL burden in 48 Asian countries and territories between 1990 and 2021

In 2021, China had the highest number of AF/AFL cases among the 48 countries and regions in Asia, with a prevalence of 10,775,720.75 (95% UI: 8,531,627.04-14,014,035.77), an incidence of 916,180.08 (95% UI: 707,383.77-1,201,380.79), 64,728.22 (95% UI: 51,764.98-77,729.27) deaths, and 1,653,116.54 (95% UI: 1,303,681.18-2,056,459.47) DALYs (Supplemental Figure 2, Supplemental Tables 2 to 5). In terms of age-standardized disease burden, Israel had the highest ASPR (1,155.51; 95% UI: 958.33-1,312.39), ASIR (91.98; 95% UI: 75.23-105.16) and age-standardized DALYs rate (148.81; 95% UI: 117.35-184.36), while Cyprus had the highest ASMR (8.36; 95% UI: 6.79-9.85) (Figure 1, Supplemental Tables 2 to 5). Meanwhile, in the vast majority of countries, the burden of AF/AFL in 2021 remained stable or increased compared with 1990 (Supplemental Tables 2 to 5).

**Figure 1:**
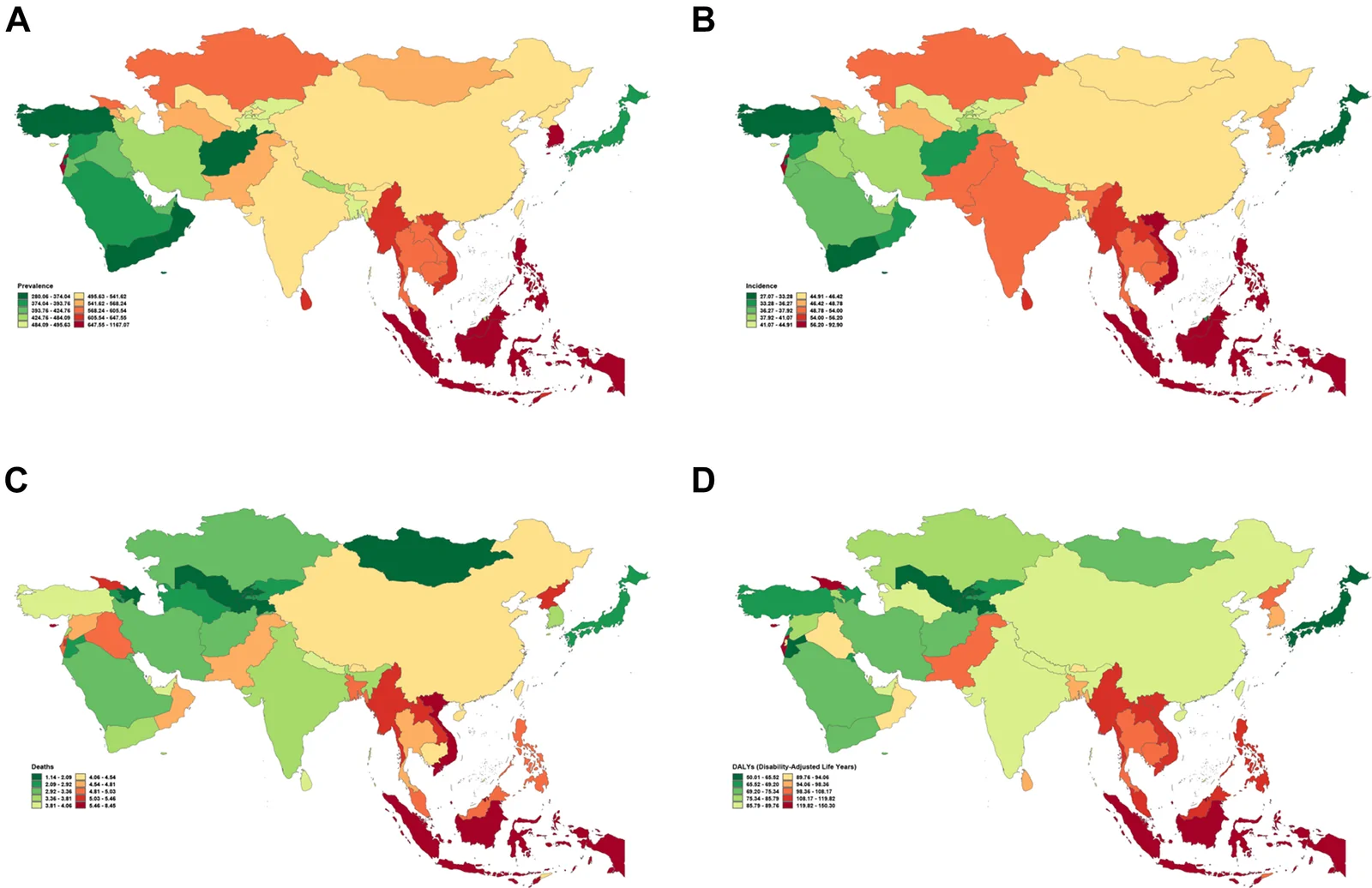
Geographic Distribution of AF/AFL Burden in Asia, 2021 (A) ASPR, (B) ASIR, (C) ASMR, and (D) age-standardized DALYs rate of AF/AFL across Asia in 2021. All data were obtained from the open database of the Global Burden of Disease study in the Global Health Data Exchange.The disease burden of AF/AFL varied across different regions in 2021. AF/AFL = atrial fibrillation and flutter; ASIR = age-standardized incidence rate; ASMR = age-standardized mortality rate; ASPR = age-standardized prevalence rate; DALYs = disability-adjusted life years.

### Disease burden of AF/AFL by sex and age in Asia

With regard to gender differences in the burden of AF/AFL in 2021, ASRs for most indicators in the Asian region were higher for males than for females (Table 1, Figure 2). The ASPR was 589.46 (95% UI: 467.45-762.42) for males and 475.28 (95% UI: 374.54-611.44) for females; the ASIR was 48.66 (95% UI: 37.83-63.90) for males and 44.32 (95% UI: 33.87-59.02) for females; and the age-standardized DALYs rate was 93.05 (95% UI: 76.06-113.59) for males and 83.12 (95% UI: 66.48-101.85) for females. Only for ASMR was the rate slightly higher in females (3.86; 95% UI: 3.05-4.51) than in males (3.78; 95% UI: 3.29-4.32). Based on the PC from 1990 to 2021, the increase in ASPR among males (0.05, 95% UI: 0.02-0.08) was higher than that among females (0.03, 95% UI: 0.02-0.05), while the PC for ASIR, ASMR, and age-standardized DALYs rate showed no significant differences between the sexes (Table 1). Over the 30-year period, the disease burden of AF/AFL in males was significantly higher than that in females in terms of ASPR, ASIR, and age-standardized DALYs rate, while it was slightly lower in terms of ASMR (Supplemental Figure 3).

**Figure 2:**
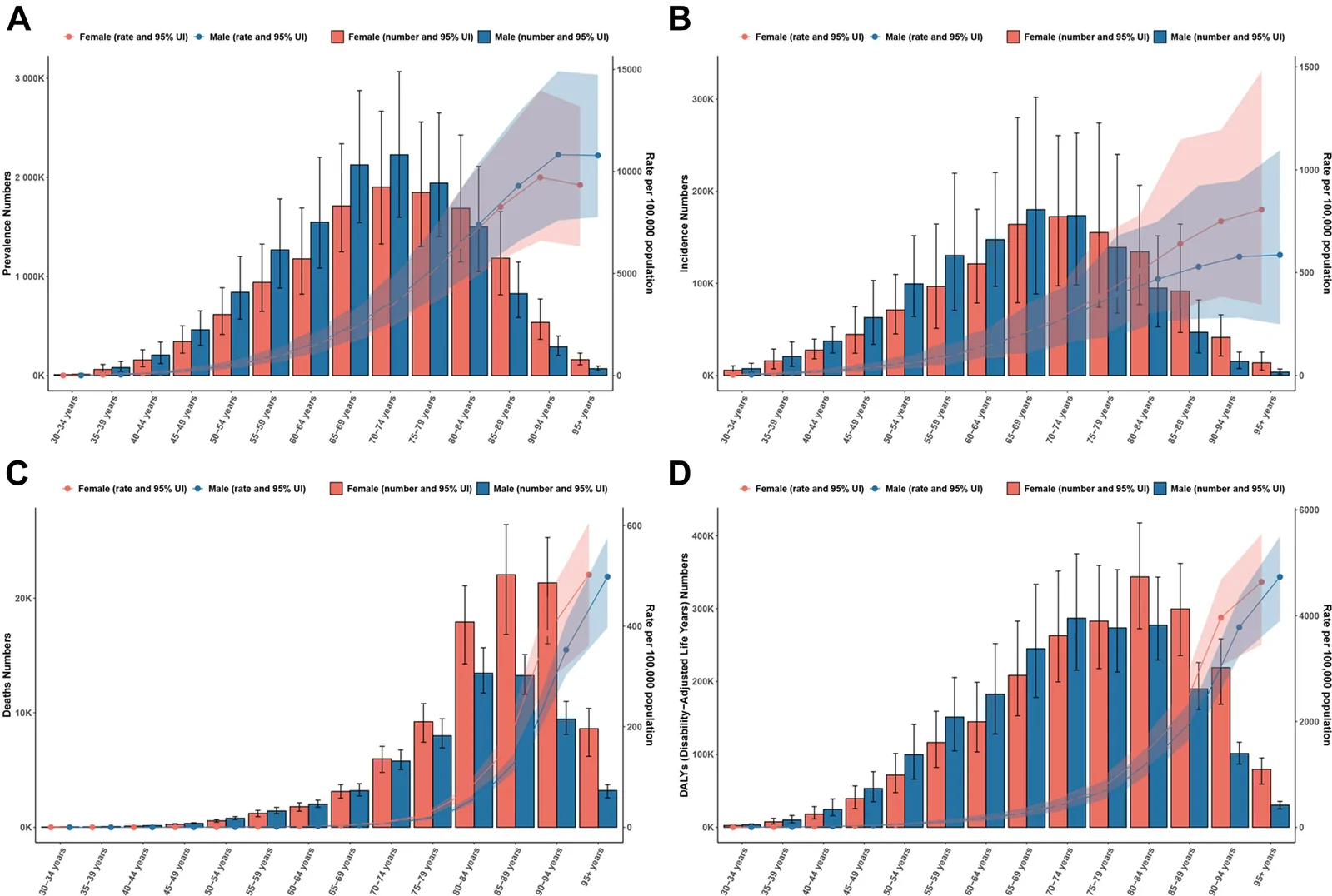
Age and Sex Disparities AF/AFL Burden in Asia, 2021 Age- and sex-based disparities in the (A) prevalence, (B) incidence, (C) mortality, and (D) DALYs of AF/AFL in Asia in 2021. All data were obtained from the open database of the Global Burden of Disease study in the Global Health Data Exchange. Disease burden was positively correlated with age. Males exhibited higher burden than females in terms of prevalence, incidence and DALYs, while elderly females were also identified as a high-risk population. AF/AFL = atrial fibrillation and flutter; DALYs = disability-adjusted life years; UI = uncertainty interval.

In 2021, the burden of AF/AFL in Asia showed a marked age-dependent increase, with prevalence, incidence, mortality, and DALYs all rising sharply among those aged ≥60, peaking in the 75-84 age group before gradually declining (Figure 2). The burden of disease was predominantly borne by males, although it was slightly higher among females in the very elderly group (aged ≥80 years). Older females constituted a high-risk group with poorer outcomes, and the overall burden of disease was highly concentrated in the elderly population. This highlights the necessity of early screening and intervention for older adults in Asia, particularly for very elderly females.

### Joinpoint analysis

Based on joinpoint regression analysis, the disease burden of AF/AFL in Asia from 1990 to 2021 exhibited complex, phased patterns of change. Figure 3 illustrates the temporal trends in ASRs for each indicator and the corresponding inflection points. The ASPR showed an overall upward trend (AAPC = 0.159%; 95% CI: 0.124-0.194, *P* < 0.001) (Table 1), and its evolution can be divided into 6 phases. From 1990 to 1993, there was a significant decline (APC = −0.700%, 95% CI: −0.829 to −0.570), followed by a narrowing of the decline from 1993 to 2000 (APC = −0.295%, 95% CI: −0.350 to −0.240); a shift to a significant increase from 2000 to 2004 (APC = +1.075%, 95% CI: 0.943-1.207); a slight decline from 2004 to 2009 (APC = −0.133%, 95% CI: −0.226 to −0.041); from 2009 to 2014, it resumed slow growth (APC = +0.082%, 95% CI: −0.010 to 0.175), and from 2014 to 2021, the rate of increase accelerated significantly (APC = +0.721%, 95% CI: 0.666-0.777) (Figure 3A). The ASIR also exhibited dynamic changes (AAPC = 0.067%; 95% CI: 0.033-0.100, *P* < 0.001) (Table 1). It showed a downward trend from 1990 to 1992 (APC = −0.733%, 95% CI: 0.936 to −0.531), with the rate of decline slowing from 1992 to 1999 (APC = −0.310%, 95% CI: −0.364 to −0.255); it then shifted to a significant increase from 1999 to 2004 (APC = +0.531%, 95% CI: 0.439-0.623), declined again from 2004 to 2009 (APC = −0.310%, 95% CI: −0.400 to −0.219); leveled off from 2009 to 2014 (APC = +0.036%, 95% CI: −0.055 to 0.127); and increased at an accelerated rate from 2014 to 2021 (APC = +0.631%, 95% CI: −0.576 to 0.685) (Figure 3B). The ASMR remained relatively stable overall but showed a slight upward trend (AAPC = 0.173%; 95% CI: 0.062-0.283, *P* < 0.001) (Table 1). It rose significantly between 1990 and 1995 (APC = +1.025%, 95% CI: 0.699-1.351), increased at a more moderate rate between 1995 and 2001 (APC = +0.200%, 95% CI: −0.123 to 0.524), and then declined between 2001 and 2007 (APC = −0.747%, 95% CI: −1.067 to −0.426), followed by a return to slow growth from 2007 to 2021 (APC = +0.251%, 95% CI: 0.161-0.340) (Figure 3C). The age-standardized DALYs rate showed a fluctuating upward trend (AAPC = 0.109%; 95% CI: 0.065-0.153, *P* < 0.001) (Table 1). From 1990 to 2003, it showed a slight increase (APC = +0.074%, 95% CI: 0.025-0.123), followed by a slight decline from 2004 to 2012 (APC = −0.173%, 95% CI: −0.269 to −0.078), and a significant acceleration in growth from 2012 to 2021 (APC = +0.441%, 95% CI: 0.345-0.537) (Figure 3D).

**Figure 3:**
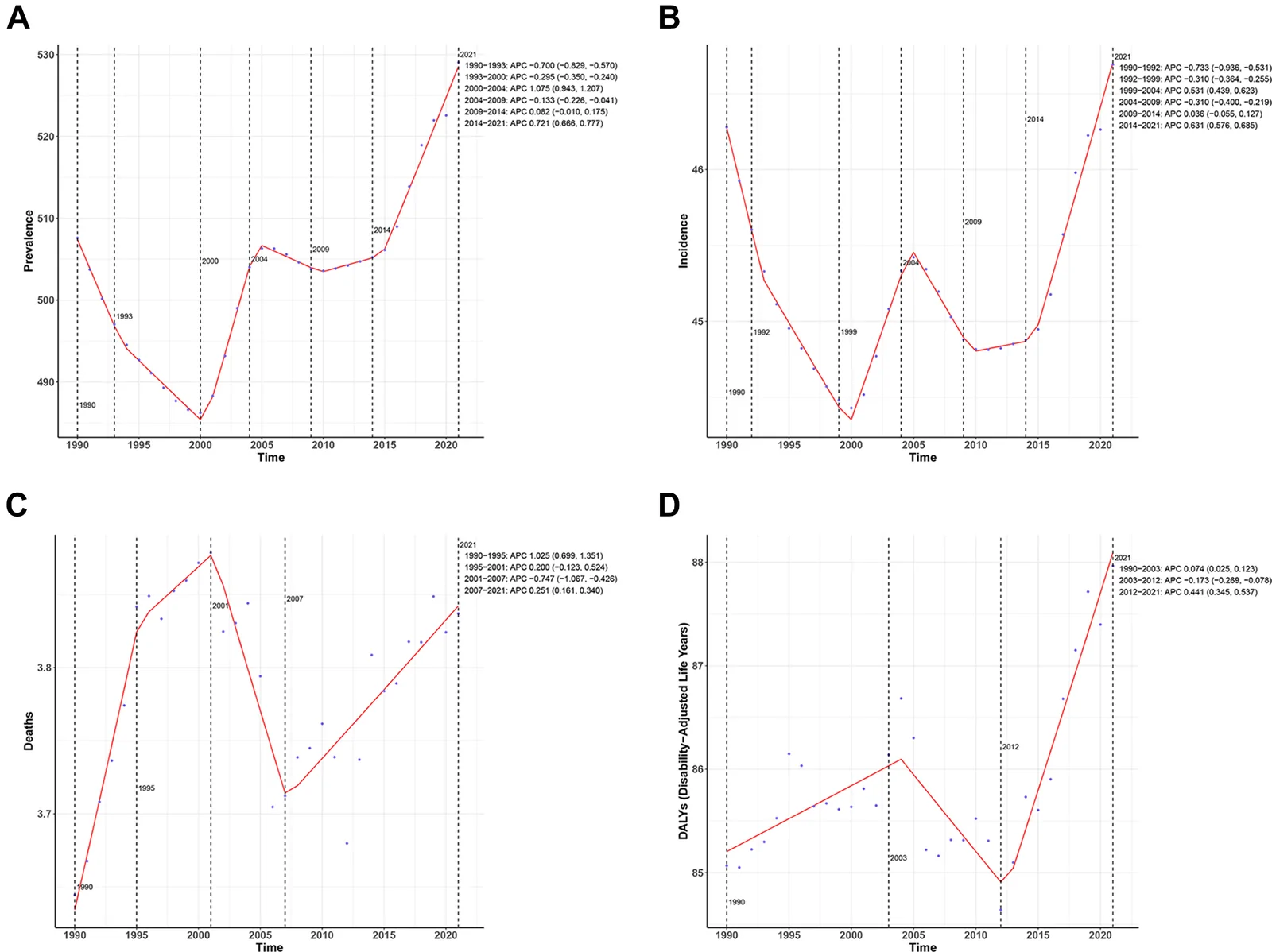
Joinpoint Regression Analysis of AF/AFL Burden in Asia, 1990 to 2021 (A) ASPR, (B) ASIR, (C) ASMR, and (D) age-standardized DALYs rate of AF/AFL burden trends in Asia, 1990 to 2021. All data were obtained from the open database of the Global Burden of Disease study in the Global Health Data Exchange, and analyses were performed using the `segmented' package in R version 4.4.2. All indicators fluctuated considerably during the study period but showed an overall increasing trend in 2021 compared with 1990, particularly after 2014. APC = annual percentage change; Other abbreviations as in Figure 1.

In summary, the phased inflection points and variations in rates of change observed in the time series of the aforementioned indicators reflect that the evolution of the disease burden of AF/AFL is not a simple linear process. It is worth noting that after 2014, all indicators generally entered a period of accelerated growth; the clinical and public health implications of this trend warrant further attention. The results of the joinpoint analysis for various subregions in Asia are presented in Table 1 and Supplemental Figure 4.

### Decomposition analysis

A decomposition analysis across 3 dimensions revealed that population growth is the primary driver of the rising disease burden of AF/AFL across Asian regions, accounting for over 50% of the contribution in most regions (Figure 4, Supplemental Table 6). In the high-income Asia Pacific region, this factor contributed 127.92% and 99.68% to incidence and prevalence, respectively. The impact of population aging was particularly pronounced in East, South, and Southeast Asia. In terms of mortality and DALYs, the contribution of population aging in East Asia reached 62.5% and 45.07% respectively, indicating a significant trend towards the concentration of the disease burden among the elderly population in this region. In contrast, the contribution of epidemiological changes varied across regions. In high-income Asia Pacific countries, the contribution was negative for all indicators, suggesting potential progress in disease prevention, control, and clinical management in this region. Conversely, in Central Asia, the contribution of epidemiological changes to prevalence was all positive. Overall, the increasing burden of AF/AFL in Asia is primarily attributable to the combined effects of population growth and aging, although the key drivers and trends in epidemiological changes vary slightly across regions.

**Figure 4:**
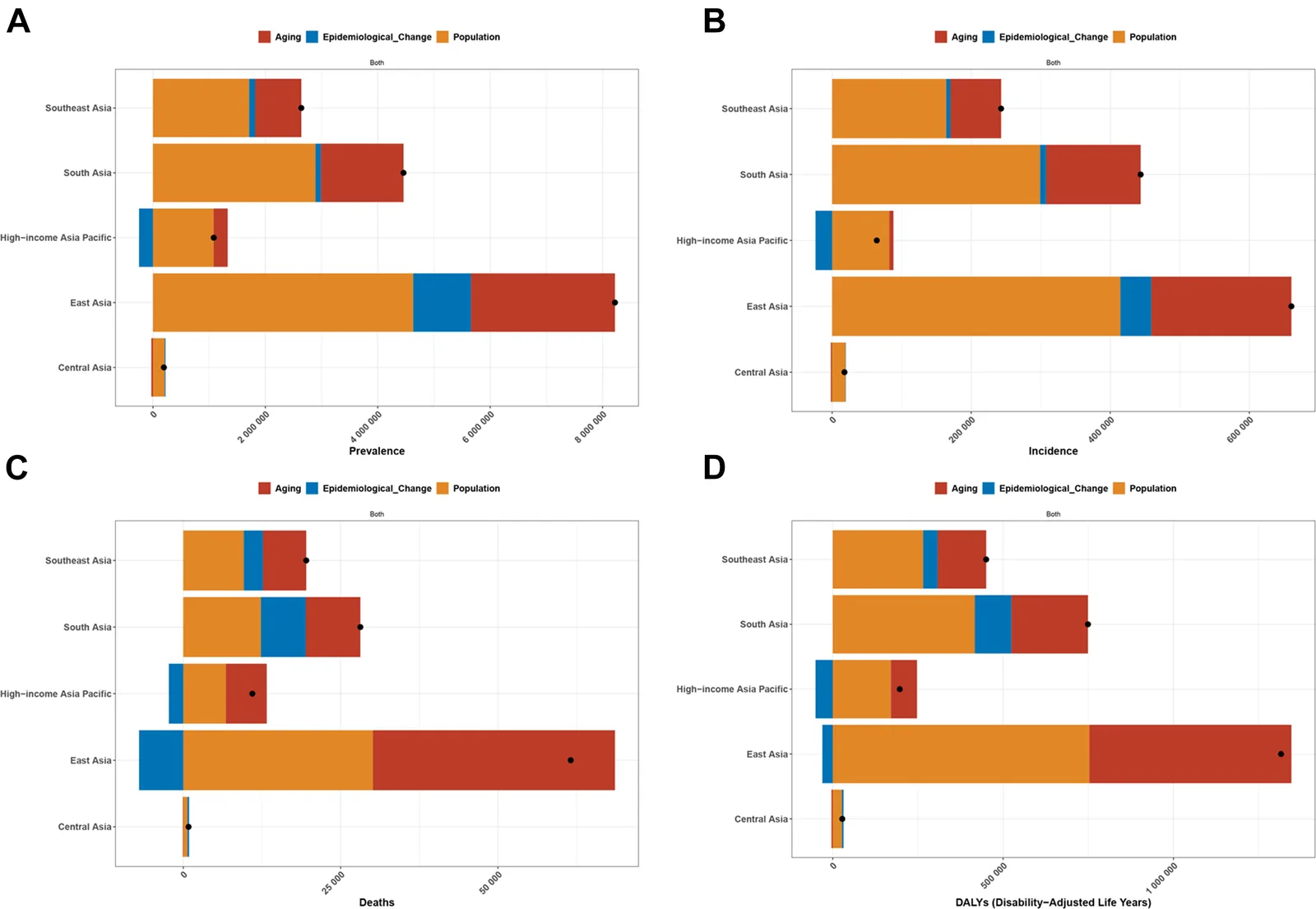
Decomposition Analysis of AF/AFL Burden in Asian Subregions (A) Decomposition analysis of (A) prevalence, (B) incidence, (C) mortality, and (D) DALYs in subregions of Asia. All data were obtained from the open database of the Global Burden of Disease study in the Global Health Data Exchange. Population growth was the primary driver of the increasing disease burden across subregions of Asia. Abbreviations as in Figure 1.

To verify the driver shifts corresponding to the 2014 inflection point, we performed period-specific decomposition analyses for 1990-2013 and 2014-2021 (Supplemental Figures 5 and 6, Supplemental Tables 7 and 8). Distinct differences were found between the two periods: Epidemiological change reversed from a burden-mitigating factor (with negative contributions in East Asia and high-income Asia Pacific) in the earlier period to a positive driver of growth after 2014; the relative contribution of population aging rose consistently across most regions and became the leading driver of mortality and DALYs in East Asia in 2014 to 2021; population growth remained a stable positive driver but declined in relative weight.

### Risk factors of AF/AFL in Asia

Trend analysis of risk factors showed that, between 1990 and 2021, high SBP consistently remained the risk factor contributing most significantly to AF/AFL-related deaths in Asia, with its burden remaining stable at a high level over the course of more than 3 decades. The contribution of high BMI exhibited the most pronounced upward trend, with its burden more than tripling, making it the fastest-growing risk factor. The attributable burden of smoking has shown a continuous downward trend, while the attributable values for high-sodium diet and high alcohol consumption have both risen slowly (Figure 5A, Supplemental Table 9). In terms of DALYs, the ranking and trends of the various risk factors are largely consistent with the mortality analysis. High SBP remains the leading factor, the increase in high BMI is particularly pronounced, and the attributable burden of smoking also shows a downward trend (Figure 5B, Supplemental Table 9).

**Figure 5:**
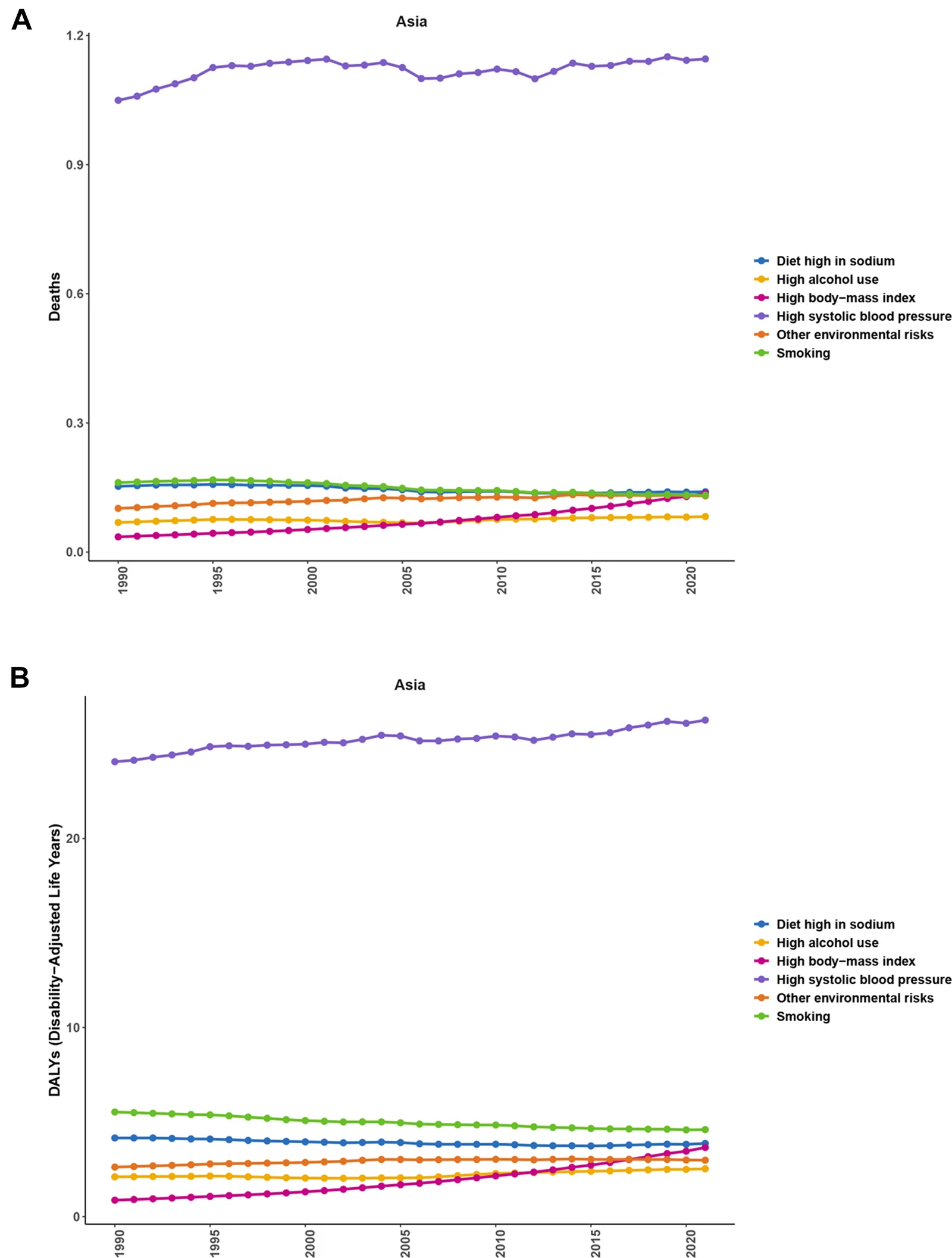
Risk Factor–Attributable AF/AFL Burden in Asia, 1990 to 2021 (A) Changes in risk factors associated with (A) ASMR and (B) age-standardized DALYs rate from 1990 to 2021. All data were obtained from the open database of the Global Burden of Disease study in the Global Health Data Exchange. High systolic blood pressure was the leading risk factor for AF/AFL, while high body mass index showed a continuous increase in recent years. AF/AFL = atrial fibrillation and flutter; ASMR = age-standardized mortality rate; DALYs = disability-adjusted life years.

### AF/AFL burden projections to 2040

Forecast data indicates that between 2022 and 2040, the prevalence, incidence, mortality, and DALYs associated with AF/AFL in Asia will all show a sustained upward trend (Figure 6, Supplemental Table 10). The prevalence is projected to rise from approximately 25,739,093 cases (95% UI: 20,431,798-33,586,165) in 2021 to approximately 58,536,068.26 cases (95% UI: 34,456,302.21-99,443,964.31) in 2040, more than doubling. The increase is particularly pronounced among females, rising from approximately 12,337,150 cases (95% UI: 9,769,353-15,906,473) to approximately 40,884,245.33 cases (95% UI: 15,839,428.11-105,529,158.32), while among males, the figure is expected to rise from approximately 13,401,943 cases (95% UI: 10,638,584-17,397,194) to approximately 27,529,972.21 cases (95% UI: 16,255,928.81-46,622,950.84). The incidence of the disease follows a similar growth trajectory, with the total rising from approximately 2,316,760 cases (95% UI: 1,795,505-3,052,653) to approximately 4,874,796.55 cases (95% UI: 3,126,339.98-7,601,105.96), and the increase among females being significantly higher than that among males. In terms of deaths, the overall figure rises from approximately 153,470 (95% UI: 127,841-172,464) to approximately 354,762.05 (95% UI: 316,559.11-397,575.38); deaths among females rise from approximately 92,292 (95% UI: 73,137-107,767) to approximately 212,928.36 (95% UI: 194,709.53-232,851.90), while deaths among males rise from approximately 61,178 (95% UI: 53,814-69,904) to approximately 141,369.80 (95% UI: 126,065.49-158,532.04). DALYs increased from approximately 4,026,948 person-years (95% UI: 3,269,416-4,982,293) to approximately 8,553,970.34 person-years (95% UI: 7,129,782.62-10,262,642.28), with females consistently accounting for a higher proportion of this burden. At the subregional level, with the exception of the high-income Asia Pacific region, which displayed a relatively moderate growth trajectory, the absolute burden in all other subregions is showing a clear upward trend (Supplemental Figure 7). Supplemental Figures 8 to 11 consistently demonstrate that the growth rate of AF/AFL burden has been higher in females than in males in recent years. Accordingly, although age-standardized AF/AFL burden remains higher in males at present, the absolute burden among females is projected to follow an accelerating increasing trend in the future.

**Figure 6:**
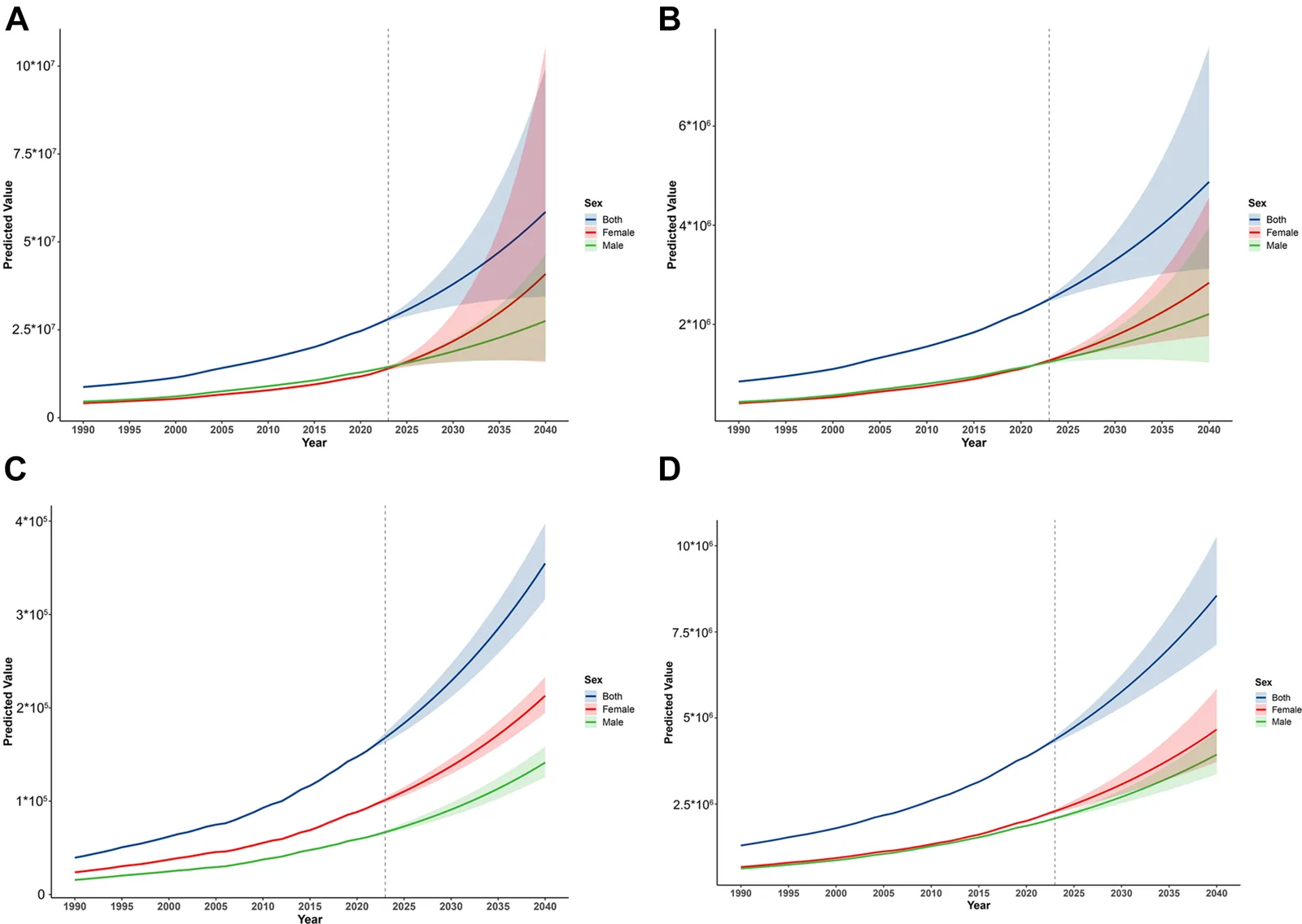
Projections of AF/AFL in Asia from 2022 to 2040 Projections of the (A) prevalence, (B) incidence, (C) mortality, and (D) DALYs of AF/AFL in Asia up to 2040. All data were obtained from the open database of the Global Burden of Disease study in the Global Health Data Exchange, and statistical analyses were performed using the ARIMA model in R version 4.4.2. The absolute burden of AF/AFL in Asia will continue to rise from 2022 to 2040. AF/AFL = atrial fibrillation and flutter; ARIMA = auto-regressive integrated moving average. DALYs = disability-adjusted life years.

## Discussion

Based on GBD 2021 data, this study provides the first systematic assessment of the disease burden of AF/AFL across 48 countries and regions in Asia between 1990 and 2021, revealing substantial heterogeneity in the distribution, temporal trends, and drivers of the disease burden across the region (Central Illustration). These findings update the epidemiological data on AF/AFL in Asia while providing evidence-based support for the formulation of targeted regional prevention and control strategies.

**Central Illustration:**
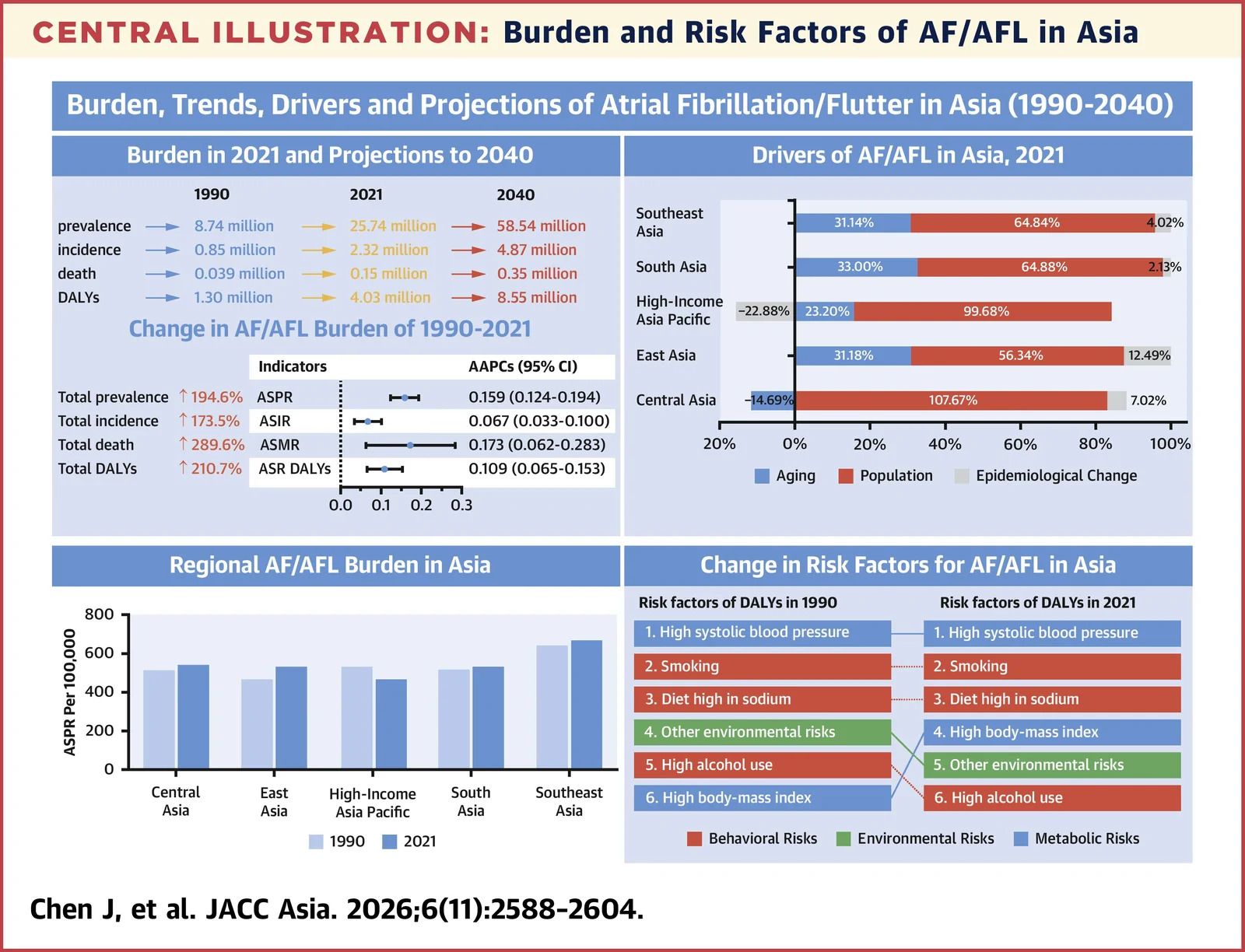
Burden and Risk Factors of AF/AFL in Asia The data used for all analyses were derived from the Global Burden of Disease study 2021. From 1990 to 2021, the number of prevalence, incidence, death and DALYs of AF/AFL increased in Asia, and will continue to increase through 2040. A decreasing trend in ASPR from 1990 to 2021 was observed solely in the high-income Asia Pacific region, in contrast to the increasing trends seen across all other Asian subregions. Population growth is the primary driving factor behind the rising burden of AF/AFL in Asia. High systolic blood pressure remains a major risk factor for AF/AFL, whereas the impact of high body mass index has been steadily rising in recent years. AF/AFL = atrial fibrillation and flutter; ASIR = age-standardized incidence rate; ASMR = age-standardized mortality rate; ASPR = age-standardized prevalence rate; ASR = age-standardized rate; DALYs = disability-adjusted life years.

The burden of AF/AFL in Asia exhibits significant regional variations, which are closely associated with disparities in economic development and health care capacity. In 2021, Southeast Asia showed the highest age-standardized disease burden across all indicators, whereas the high-income Asia Pacific region demonstrated a declining trend. In Southeast Asia, rapid economic transition has been accompanied by a rising prevalence of metabolic risk factors,^34^ uneven health care resource distribution,^35^ and low anticoagulant coverage in some countries,^36^ collectively contributing to the heavy disease burden. In contrast, the high-income Asia-Pacific region benefits from well-established primary care,^37^ widespread cardiovascular screening,^38^ and high adoption of novel oral anticoagulants,^39^^,^^40^ supporting effective prevention and control of AF/AFL.^41^^,^^42^ Notably, similar favorable trends have been observed for other major cardiovascular diseases, including ischemic heart disease and heart failure. For instance, Goh et al^43^ projected that by 2050, high-income Asia Pacific countries will maintain the lowest age-standardized cardiovascular mortality in Asia, reflecting long-term investment in health care infrastructure and comprehensive prevention. Likewise, Feng et al^44^ reported that improved control of hypertension and ischemic heart disease could further reduce the heart failure burden in these regions. Despite the overall downward trend in most high-income Asia Pacific countries, heterogeneity remains given differences in health care organization, screening implementation, risk factor management, and cardiovascular prevention networks. These observations indicate that economic growth alone is insufficient to manage AF/AFL burden,^45^^,^^46^ and a systematic cardiovascular disease prevention network is critical for effective control.^40^^,^^47^

Joinpoint regression analysis revealed a nonlinear pattern in the evolution of the burden of AF/AFL. It is worth noting that the ASIR experienced a brief but significant rise between 1999 and 2004, followed by a plateau, before accelerating again after 2014. These phased changes may reflect the dynamic interplay of multiple factors across different periods, including fluctuations in exposure to risk factors, increased case detection rates because of the widespread adoption of diagnostic technologies,^40^^,^^48^ and the corrective effect of improved disease registration systems on statistical data.^39^^,^^48^ All of these factors may have exerted varying degrees of influence on incidence trends at different stages. In particular, the comprehensive acceleration observed since 2014 corresponds with the rapid spread of metabolic risk factors, especially overweight and obesity during the same period.^49^, ^50^, ^51^ This suggests that existing public health interventions may face significant challenges in curbing the growing burden of disease, necessitating a rethinking of current prevention and control strategies.

A decomposition analysis revealed that demographic changes play a dominant role in the increase in the burden of disease, but the driving patterns vary significantly across different regions. In high-income Asia Pacific regions, population growth alone would have been responsible for 127.92% and 99.68% of the increases in incidence and prevalence, respectively, suggesting that the actual burden increase would have been far larger without the mitigating effects of epidemiological changes. The negative contribution of epidemiological changes further indicates that effective prevention and clinical management have partially offset the burden increase attributable to population growth.^52^^,^^53^ This pattern contrasts markedly with that observed in East Asia, East and Southeast Asia, and East, South, and Southeast Asia, with population aging contributing as much as 62.5% and 45.07% to the increase in mortality and DALYs in East Asia, respectively, reflecting the structural pressure these regions face because of the high concentration of the disease burden among the elderly population.^54^ From a public health perspective, the experience of high-income Asia Pacific regions indicates that systematic prevention and control measures can effectively stabilize disease burden despite sustained population growth; whereas East Asia and South Asia may need to prioritize the development of targeted geriatric disease management frameworks to mitigate challenges associated with rapid population aging. The period-specific decomposition analysis further revealed that the relative weight of drivers has shifted substantially since 2014. The weakening protective effect of epidemiological change and the growing contribution of aging jointly explain the accelerated growth of AF/AFL burden observed after 2014.

The results of the risk factor attribution analysis reveal that the risk factor profile for AF/AFL is gradually shifting from the traditional model dominated by hypertension alone to one characterized primarily by the accumulation of multiple metabolic risk factors. High SBP has consistently been the primary risk factor for AF/AFL-related mortality, with its attributable burden remaining stable at a high level over the past 3 decades;^19^ this finding is consistent with the epidemiological characteristics of hypertension in Asia, high prevalence, and poor control rates.^55^ More notably, the attributable burden of high BMI has more than tripled over the past 3 decades, representing the most significant increase among all risk factors. This trend coincides with the rapid rise in obesity rates across Asia,^56^ further confirming that the structure of AF/AFL risk factors is undergoing a substantial shift. This transformation underscores the need for a corresponding evolution in prevention strategies. The continued decline in the burden associated with smoking may reflect the phased success of tobacco control policies in some parts of Asia;^57^^,^^58^ however, this positive change is uneven across regions, with areas outside the high-income Asia Pacific region still exhibiting significant gaps in the coverage or intensity of tobacco control measures.^59^ These findings offer dual implications for clinical practice and public health strategies: On the one hand, hypertension management should remain a core component of primary prevention for AF/AFL, with continued efforts to strengthen the implementation and enforcement of blood pressure control measures; on the other hand, lifestyle interventions targeting overweight and obesity urgently need to be incorporated into the strategic priorities of regional cardiovascular disease prevention and control to address the further rise in the disease burden in the future.

Analysis stratified by sex and age reveals that although men have higher disease burden indicators than women in most cases, elderly women exhibit a higher burden in the very old age group, and projections indicate that the increase in the burden among women will significantly outpace that among men by 2040. This phenomenon may be linked to the fact that a higher proportion of women are diagnosed at a later age. According to a nationwide Finnish cohort study, women were diagnosed with AF/AFL at a significantly older age than men (76.6 ± 11.8 years vs 68.9 ± 13.4 years).^60^ It is worth noting that, as women face a higher risk of stroke and bleeding during anticoagulant therapy,^61^ this leads to more conservative clinical decision-making. In particular, older women often have lower coverage and adherence to anticoagulation and rhythm control therapies.^60^ Furthermore, elderly women often experience multiple complications, including hypertension, diabetes, and heart failure; this state of multimorbidity complicates disease management and increases the risk of adverse outcomes.^62^^,^^63^ From a clinical practice perspective, current guidelines for AF/AFL management lack sufficient emphasis on gender-specific strategies, particularly those targeting elderly women.^64^^,^^65^ Future research should further explore optimal management strategies for elderly women with AF/AFL, including individualized decision-making regarding anticoagulation therapy, comprehensive management of complications, and the optimization of multidisciplinary collaboration models.

Predictive analysis indicates that the burden of AF/AFL in Asia will continue to rise between 2022 and 2040. The total number of cases is projected to rise from approximately 25.74 million to around 58.54 million, more than doubling over the period. This trend will place sustained and significant pressure on health care systems across Asia. It is worth noting that the increase in disease burden is markedly more pronounced among females than among males. This accelerating growth trajectory in females may reflect not only a shift in the underlying epidemiology of disease incidence, but also a progressive narrowing of sex disparities and an improvement in diagnostic parity. This finding further underscores the need to incorporate gender differences into prevention and control strategies. From a regional perspective, the absolute burden of AF/AFL has exhibited a substantial upward trend across most Asian subregions, with the only exception being the high-income Asia Pacific region, where the increase has been relatively modest. Such divergent trends imply that interregional disparities in disease burden are likely to widen further in the future. Therefore, the implementation of region-targeted interventions represents a crucial strategy for the prevention and control of AF/AFL across Asia.

### Study limitations

Firstly, the quality and richness of underlying source data vary substantially across the 48 included countries and territories, which may introduce estimation biases in regions with weak data infrastructure. High-income Asia Pacific economies are supported by well-established vital registration systems and high-quality primary clinical datasets, such that their burden estimates are predominantly driven by observed empirical data. In contrast, some countries in Central and Southeast Asia have sparser source data inputs, and their estimates rely more heavily on Bayesian spatio-temporal imputation and model extrapolation. To mitigate the impact of data heterogeneity, this study used the 95% uncertainty interval provided by GBD and conducted aggregated analyses at the subregional level to enhance the robustness of the estimates. Nevertheless, the uneven evidentiary quality may affect the accuracy of burden estimates and decomposition results; correspondingly, the reliability of future projections also differs across regions, with greater uncertainty for data-sparse settings. Secondly, because of the inherent limitations of the GBD data, this study was unable to distinguish between the burden of paroxysmal AF/AFL and that of persistent AF, despite significant differences in clinical management strategies and prognosis between these types of AF. Future research should use multicenter prospective cohorts in Asia to further explore the interactions of region-specific risk factors and conduct cost-effectiveness analyses of intervention strategies tailored to different population characteristics, thereby addressing the aforementioned gaps to inform more targeted prevention strategies. Third, caution is warranted when interpreting the breakpoints identified via joinpoint regression. These inflection points may be influenced by stochastic fluctuations, heterogeneity in data sources, or modeling adjustments across GBD study cycles. Interpretation of year-specific inflections should be contextualized against potential shifts in underlying clinical or policy drivers to avoid misclassifying short-term fluctuations as fundamental epidemiological changes.

## Conclusions

This study assessed the burden of AF/AFL across 48 countries and regions in Asia between 1990 and 2021. The results indicate a significant upward trend, marked regional heterogeneity, and an ongoing shift in the risk factor profile. In the face of this persistently rising disease burden, Asian countries need to formulate region-specific prevention and control strategies, in order to effectively reduce the impact of these conditions.

## Funding Support and Author Disclosures

The authors have reported that they have no relationships relevant to the contents of this paper to disclose.
