# Supplementary material for: Epidemiological Analysis and 2040 Projections of Atrial Fibrillation and Flutter in Asia
Source: JACC Asia. 2026 Aug 20;6(11):2588–604. doi: 10.1016/j.jacasi.2026.06.037 (PMC13750910; doi:10.1016/j.jacasi.2026.06.037)
Supplement: Supplementary file 1 [file mmc1.docx]

**Epidemiological Analysis and 2040 Projections of Atrial Fibrillation and Flutter in Asia**

Jing Chen, MM^a,b^, Shunyu Yao, MM^a,b^, Zhongshan Xiong, MM^a,b^, Linfeng Xie, MM^a,b^, Yuanzhu Li, MM^a,b^, Gregory Y. H. Lip, MD, P_H_D^c,d,e,*^, Bi Huang,MD, P_H_D^a,b,c*^

^a^Department of Cardiovascular Medicine, Cardiovascular Research Center, The First Affiliated Hospital of Chongqing Medical University, Chongqing, 400016, China

^b^Cardiovascular Disease Laboratory, Chongqing Medical University, Chongqing, China

^c^Liverpool Centre for Cardiovascular Science at University of Liverpool, Liverpool John Moores University, and Liverpool Heart & Chest Hospital, Liverpool, United Kingdom

^d^Department of Clinical Medicine, Aalborg University, Aalborg, Denmark

^e^Medical University of Bialystok, Bialystok, Poland

***joint senior authors**

**Correspondence to:**

Gregory Y. H. Lip, Liverpool Centre for Cardiovascular Science at University of Liverpool, Liverpool John Moores University, and Liverpool Heart & Chest Hospital, Liverpool, United Kingdom. Email: Gregory.Lip@liverpool.ac.uk

Bi Huang, Department of Cardiology, The First Affiliated Hospital of Chongqing Medical University, NO.1 Youyi Road, Yuzhong District, Chongqing, 400016, China. Email: [204194@hospital.cqmu.edu.cn](mailto:204194@hospital.cqmu.edu.cn)

****Supplementary Table of Contents****

[Supplemental Figure 1: Temporal Trends of AF/AFL Across Asian Sub-regions (1990–2021)	3](#_Toc28283)

[Supplemental Figure 2: Numbers of AF/AFL Across Asia in 2021	4](#_Toc21600)

[Supplemental Figure 3: Sex-Specific AF/AFL Trends in Asia (1990–2021)	5](#_Toc22613)

[Supplemental Figure 4: AAPCs of AF/AFL Prevalence, Incidence, Mortality, and DALYs Across Asia, 1990-2021	6](#_Toc15928)

[Supplemental Figure 5: Decomposition Analysis of AF/AFL Burden in Asian Sub-regions, 1990-2013	7](#_Toc16187)

[Supplemental Figure 6: Decomposition Analysis of AF/AFL Burden in Asian Sub-regions, 2014-2021	8](#_Toc19550)

[Supplemental Figure 7: Projected AF/AFL Burden in Asian Sub-regions to 2040	9](#_Toc15330)

[Supplemental Figure 8: Joinpoint Regression Analysis of the Burden of AF/AFL Among Male with Crude Rate in Asia, 1990-2021	10](#_Toc4667)

[Supplemental Figure 9: Joinpoint Regression Analysis of the Burden of AF/AFL Among Female with Crude Rate in Asia, 1990-2021	11](#_Toc9075)

[Supplemental Figure 10: Joinpoint Regression Analysis of the Burden of AF/AFL Among Male with Age-standardized Rate in Asia, 1990-2021	12](#_Toc27183)

[Supplemental Figure 11: Joinpoint Regression Analysis of the Burden of AF/AFL Among Female with Age-standardized Rate in Asia, 1990-2021	13](#_Toc16705)

[Supplementary Table S1: List of 48 Countries and Territories in Asia.	14](#_Toc10988)

[Supplementary Table S2: Prevalence of AF/AFL in Asia in 1990 and 2021.	15](#_Toc3891)

[Supplementary Table S3: Incidence of AF/AFL in Asia in 1990 and 2021.	17](#_Toc29881)

[Supplementary Table S4: Death of AF/AFL in Asia in 1990 and 2021.	19](#_Toc27276)

[Supplementary Table S5: DALYs for AF/AFL in Asia in 1990 and 2021.	21](#_Toc15759)

[Supplementary Table S6: The decomposition analysis of AF/AFL in Asia, 1990-2021.	23](#_Toc28671)

[Supplementary Table S7: The decomposition analysis of AF/AFL in Asia, 1990-2013.	24](#_Toc15224)

[Supplementary Table S8: The decomposition analysis of AF/AFL in Asia, 2014-2021.	25](#_Toc16672)

[Supplementary Table S9: Risk factors of AF/AFL in Asia, 1990 to 2021.	26](#_Toc1692)

[Supplementary Table S10. Global disease burden prediction for AF/AFL in Asia to 2040.	28](#_Toc18108)

# **Supplemental Figure 1: Temporal Trends of AF/AFL Across Asian Sub-regions (1990–2021)**

(A) ASPR of AF/AFL across Asian sub-regions from 1990 to 2021. (B) ASIR of AF/AFL across Asian sub-regions from 1990 to 2021. (C) ASMR of AF/AFL across Asian sub-regions from 1990 to 2021. (D) Age‑standardized DALYs rate of AF/AFL across Asian sub-regions from 1990 to 2021. All data were obtained from the open database of the Global Burden of Disease study in the Global Health Data Exchange. The changing trends vary across different Asian sub-regions****.**** AF/AFL = atrial fibrillation and flutter; ASPR = age-standardized prevalence rate; ASIR = age-standardized incidence rate; ASMR = age-standardized mortality rate; DALYs = disability-adjusted life years.

# **Supplemental Figure 2: Numbers of AF/AFL Across Asia in 2021**

(A) Prevalence number of AF/AFL across Asia in 2021. (B) Incidence number of AF/AFL across Asia in 2021. (C) Mortality number of AF/AFL across Asia in 2021. (D) DALYs number of AF/AFL across Asia in 2021. All data were obtained from the open database of the Global Burden of Disease study in the Global Health Data Exchange. In 2021, the burden of AF/AFL across Asian countries and territories exhibited regional heterogeneity, with China and India having the highest absolute burden of the disease. Abbreviations as in Supplemental Figure 1.

**s.**

# **Supplemental Figure 3: Sex-Specific AF/AFL Trends in Asia (1990–2021)**

(A) ASPR of AF/AFL in different sex across Asia from 1990 to 2021. (B) ASIR of AF/AFL in different sex across Asia from 1990 to 2021. (C) ASMR of AF/AFL in different sex across Asia from 1990 to 2021. (D) Age‑standardized DALYs rate of AF/AFL in different sex across Asia from 1990 to 2021. All data were obtained from the open database of the Global Burden of Disease study in the Global Health Data Exchange. Over the past 30 years, males had higher ASPR, ASIR, and Age‑standardized DALYs rate than females, whereas the ASMR was slightly higher in females than in males.. Abbreviations as in Supplemental Figure 1.

# **Supplemental Figure 4: AAPCs of AF/AFL Prevalence, Incidence, Mortality, and DALYs Across Asia, 1990-2021**

(A) AAPC of AF/AFL in ASPR across Asia from 1990 to 2021. (B) AAPC of AF/AFL in ASIR across Asia from 1990 to 2021. (C) AAPC of AF/AFL in ASMR across Asia from 1990 to 2021. (D) AAPC of AF/AFL in age‑standardized DALYs rate across Asia from 1990 to 2021. All data were obtained from the open database of the Global Burden of Disease study in the Global Health Data Exchange, and analyses were performed using the `segmented` package in R version 4.4.2. The trends of these indicators show heterogeneity across Asia. AAPC = annual average percentage change; Other abbreviations as in Supplemental Figure 1.

# **Supplemental Figure 5: Decomposition Analysis of AF/AFL Burden in Asian Sub-regions, 1990-2013**

(A) Decomposition analysis of prevalence in sub-regions of Asia. (B) Decomposition analysis of incidence in sub-regions of Asia. (C) Decomposition analysis of mortality in sub-regions of Asia. (D) Decomposition analysis of DALYs in sub-regions of Asia. All data were obtained from the open database of the Global Burden of Disease study in the Global Health Data Exchange. Population growth was the primary driver of the increasing disease burden across sub-regions of Asia. Abbreviations as in Figure 1.

# **Supplemental Figure 6: Decomposition Analysis of AF/AFL Burden in Asian Sub-regions, 2014-2021**

(A) Decomposition analysis of prevalence in sub-regions of Asia. (B) Decomposition analysis of incidence in sub-regions of Asia. (C) Decomposition analysis of mortality in sub-regions of Asia. (D) Decomposition analysis of DALYs in sub-regions of Asia. All data were obtained from the open database of the Global Burden of Disease study in the Global Health Data Exchange. Population growth was the primary driver of the increasing disease burden across sub-regions of Asia. Abbreviations as in Figure 1.

# **Supplemental Figure 7: Projected AF/AFL Burden in Asian Sub-regions to 2040**

All data were obtained from the open database of the Global Burden of Disease study in the Global Health Data Exchange, and statistical analyses were performed using the ARIMA model in R version 4.4.2. By 2040, the absolute burden of AF/AFL is expected to rise in every sub-region. ARIMA = auto-regressive integrated moving average.

# **Supplemental Figure 8: Joinpoint Regression Analysis of the Burden of AF/AFL Among Male with Crude Rate in Asia, 1990-2021**

(A) Prevalence rate of AF/AFL burden among male in Asia, 1990-2021. (B) Incidence rate of AF/AFL burden among male in Asia, 1990-2021. (C) Mortality rate of AF/AFL burden among male in Asia, 1990-2021. (D) DALYs rate of AF/AFL burden among male in Asia, 1990-2021. All data were obtained from the open database of the Global Burden of Disease study in the Global Health Data Exchange, and analyses were performed using the `segmented` package in R version 4.4.2. All indicators fluctuated considerably during the study period but showed an overall increasing trend in 2021 compared with 1990, particularly after 2014. All abbreviations as in Table 1, Figure 1 and figure 3.

# **Supplemental Figure 9: Joinpoint Regression Analysis of the Burden of AF/AFL Among Female with Crude Rate in Asia, 1990-2021**

(A) Prevalence rate of AF/AFL burden among female in Asia, 1990-2021. (B) Incidence rate of AF/AFL burden among female in Asia, 1990-2021. (C) Mortality rate of AF/AFL burden among female in Asia, 1990-2021. (D) DALYs rate of AF/AFL burden among female in Asia, 1990-2021. All data were obtained from the open database of the Global Burden of Disease study in the Global Health Data Exchange, and analyses were performed using the `segmented` package in R version 4.4.2. All indicators fluctuated considerably during the study period but showed an overall increasing trend in 2021 compared with 1990, particularly after 2014. All abbreviations as in Table 1, Figure 1 and figure 3.

# **Supplemental Figure 10: Joinpoint Regression Analysis of the Burden of AF/AFL Among Male with Age-standardized Rate in Asia, 1990-2021**

(A) ASPR of AF/AFL burden among male in Asia, 1990-2021. (B) ASIR of AF/AFL burden among male in Asia, 1990-2021. (C) ASMR of AF/AFL burden among male in Asia, 1990-2021. (D) Age‑standardized DALYs rate of AF/AFL burden among male in Asia, 1990-2021. All data were obtained from the open database of the Global Burden of Disease study in the Global Health Data Exchange, and analyses were performed using the `segmented` package in R version 4.4.2. All abbreviations as in Table 1, Figure 1 and figure 3.

# **Supplemental Figure 11: Joinpoint Regression Analysis of the Burden of AF/AFL Among Female with Age-standardized Rate in Asia, 1990-2021**

(B) ASPR of AF/AFL burden among female in Asia, 1990-2021. (B) ASIR of AF/AFL burden among female in Asia, 1990-2021. (C) ASMR of AF/AFL burden among female in Asia, 1990-2021. (D) Age‑standardized DALYs rate of AF/AFL burden among female in Asia, 1990-2021. All data were obtained from the open database of the Global Burden of Disease study in the Global Health Data Exchange, and analyses were performed using the `segmented` package in R version 4.4.2. All abbreviations as in Table 1, Figure 1 and figure 3.

# Supplementary Table S1: List of 48 Countries and Territories in Asia.

| Afghanistan | Armenia | Azerbaijan | Bahrain | Bangladesh | Bhutan |
| --- | --- | --- | --- | --- | --- |
| Brunei Darussalam | Cambodia | China | Cyprus | Democratic People’s Republic of Korea | Georgia |
| India | Indonesia | Iran (Islamic Republic of) | Iraq | Israel | Japan |
| Jordan | Kazakhstan | Kuwait | Kyrgyzstan | Lao People’s Democratic Republic | Lebanon |
| Malaysia | Maldives | Mongolia | Myanmar | Nepal | Oman |
| Pakistan | Palestine | Philippines | Qatar | Republic of Korea | Saudi Arabia |
| Singapore | Sri Lanka | Syrian Arab Republic | Tajikistan | Thailand | Timor-Leste |
| Turkey | Turkmenistan | United Arab Emirates | Uzbekistan | Viet Nam | Yemen |

Supplementary Table S2: Prevalence of AF/AFL in Asia in 1990 and 2021.

|  | **Counts (95% UI, 1990)** | **ASPR 100,000**  **(95% UI, 1990)** | **Counts**  **(95% UI, 2021)** | **ASPR 100,000**  **(95% UI, 2021)** | **pc, (95% UI, 1990-2021)** | **AAPC (95% CI)** |
| --- | --- | --- | --- | --- | --- | --- |
| **Afghanistan** | 17,747.19 (13,622.39-23,160.12) | 312.82 (240.08-412.25) | 25,638.87 (19,700.85-33,184.36) | 338.76 (257.55-442.80) | 0.08 (0.04 to 0.13) | 0.263 (0.248 to 0.278) |
| **Armenia** | 12,536.09 (9,692.67-16,434.91) | 507.82 (391.11-664.63) | 24,452.33 (18,788.50-32,256.73) | 556.65 (424.75-729.78) | 0.1 (0.05 to 0.14) | 0.309 (0.293 to 0.325) |
| **Azerbaijan** | 22,683.37 (17,562.58-29,381.86) | 501.70 (385.38-655.28) | 48,767.42 (38,083.15-64,570.00) | 540.88 (415.38-701.51) | 0.08 (0.03 to 0.14) | 0.256 (0.241 to 0.271) |
| **Bahrain** | 462.32 (355.55-611.17) | 361.40 (274.60-473.49) | 2,667.30 (2,055.07-3,468.48) | 386.71 (293.82-505.55) | 0.07 (0.02 to 0.11) | 0.212 (0.192 to 0.232) |
| **Bangladesh** | 190,656.91 (146,465.15-248,663.35) | 472.02 (362.32-621.61) | 623,059.61 (480,696.35-812,960.53) | 494.70 (380.67-643.93) | 0.05 (0 to 0.09) | 0.157 (0.117 to 0.197) |
| **Bhutan** | 864.24 (668.25-1,134.39) | 467.22 (356.20-612.99) | 2,769.78 (2,170.17-3,629.62) | 492.91 (384.10-647.49) | 0.05 (0.01 to 0.1) | 0.174 (0.158 to 0.190) |
| **Brunei Darussalam** | 524.77 (411.92-677.05) | 537.28 (419.06-696.70) | 1,478.69 (1,154.82-1,909.94) | 452.03 (354.84-582.96) | -0.16 (-0.19 to -0.11) | -0.538 (-0.554 to -0.522) |
| **Cambodia** | 20,821.88 (16,350.14-27,382.21) | 562.64 (441.69-725.52) | 61,483.08 (48,621.11-81,181.92) | 578.56 (457.17-751.32) | 0.03 (0 to 0.06) | 0.089 (0.083 to 0.095) |
| **China** | 3,195,308.71 (2,518,982.68-4,168,289.54) | 457.72 (358.93-594.96) | 10,775,720.75 (8,531,627.04-14,014,035.77) | 524.00 (418.15-681.23) | 0.14 (0.11 to 0.19) | 0.455 (0.371 to 0.538) |
| **Cyprus** | 5,114.30 (3,908.10-6,514.09) | 652.09 (507.78-834.30) | 10,426.78 (8,434.26-13,170.01) | 487.17 (395.53-615.62) | -0.25 (-0.33 to -0.17) | -0.938 (-0.987 to -0.889) |
| **Democratic People's Republic of Korea** | 73,282.34 (57,655.35-93,864.52) | 534.03 (422.64-695.54) | 166,217.99 (132,256.02-214,516.11) | 532.40 (422.66-687.42) | 0 (-0.05 to 0.05) | -0.009 (-0.022 to 0.005) |
| **Georgia** | 33,800.31 (25,848.67-44,478.70) | 558.89 (425.91-728.40) | 35,411.56 (26,991.09-46,236.40) | 568.73 (436.23-738.62) | 0.02 (-0.02 to 0.06) | 0.066 (0.050 to 0.082) |
| **India** | 1,860,594.09 (1,442,729.00-2,458,704.14) | 521.35 (402.59-685.09) | 5,612,366.17 (4,314,557.96-7,360,330.37) | 533.92 (412.71-700.00) | 0.02 (0.01 to 0.03) | 0.077 (0.055 to 0.100) |
| **Indonesia** | 560,360.20 (442,857.69-730,937.44) | 700.63 (547.24-905.66) | 1,474,160.14 (1,152,712.71-1,922,563.67) | 728.20 (569.74-942.29) | 0.04 (0.02 to 0.06) | 0.127 (0.121 to 0.133) |
| **Iran (Islamic Republic of)** | 75,832.52 (58,813.80-99,095.18) | 385.97 (296.24-507.56) | 294,248.72 (230,024.19-382,165.91) | 425.39 (327.17-559.23) | 0.1 (0.09 to 0.12) | 0.293 (0.269 to 0.316) |
| **Iraq** | 26,905.91 (20,773.16-34,732.72) | 373.36 (285.05-485.80) | 78,700.64 (61,203.17-102,059.88) | 406.12 (308.47-532.03) | 0.09 (0.04 to 0.14) | 0.268 (0.257 to 0.279) |
| **Israel** | 41,616.42 (31,817.88-54,186.76) | 848.35 (656.52-1,096.61) | 147,351.72 (122,034.46-167,473.38) | 1,155.51 (958.33-1,312.39) | 0.36 (0.14 to 0.73) | 1.112 (1.061 to 1.162) |
| **Japan** | 873,569.60 (685,978.40-1,135,913.52) | 519.91 (411.63-671.77) | 1,498,702.00 (1,171,946.35-1,964,169.14) | 391.87 (310.63-511.22) | -0.25 (-0.27 to -0.23) | -0.740 (-0.877 to -0.604) |
| **Jordan** | 3,648.82 (2,816.06-4,742.92) | 354.23 (268.40-466.12) | 23,982.99 (18,450.51-31,112.02) | 396.61 (300.63-520.30) | 0.12 (0.07 to 0.18) | 0.366 (0.355 to 0.377) |
| **Kazakhstan** | 65,048.07 (50,302.62-84,806.30) | 562.81 (428.43-731.60) | 96,396.90 (74,220.61-126,066.65) | 589.25 (453.42-768.59) | 0.05 (0 to 0.1) | 0.147 (0.138 to 0.156) |
| **Kuwait** | 1,720.79 (1,336.63-2,199.10) | 369.85 (282.78-483.44) | 9,835.55 (7,696.84-12,637.98) | 408.07 (310.58-530.22) | 0.1 (0.05 to 0.15) | 0.297 (0.278 to 0.316) |
| **Kyrgyzstan** | 13,329.23 (10,299.02-17,483.68) | 478.91 (366.47-626.57) | 20,779.42 (16,255.59-27,051.82) | 491.16 (377.25-639.22) | 0.03 (-0.02 to 0.07) | 0.070 (0.052 to 0.089) |
| **Lao People's Democratic Republic** | 9,987.90 (7,850.66-13,151.90) | 587.21 (457.70-766.12) | 23,869.16 (18,912.59-31,406.31) | 605.11 (475.86-782.28) | 0.03 (-0.01 to 0.07) | 0.104 (0.096 to 0.111) |
| **Lebanon** | 6,336.08 (4,938.95-8,255.29) | 342.25 (261.21-444.60) | 24,591.08 (18,725.05-32,515.75) | 387.14 (298.49-511.08) | 0.13 (0.07 to 0.18) | 0.395 (0.381 to 0.409) |
| **Malaysia** | 52,681.85 (42,117.28-68,276.19) | 631.34 (495.34-821.09) | 174,175.72 (137,798.22-228,295.64) | 673.16 (526.92-873.05) | 0.07 (0.02 to 0.11) | 0.222 (0.201 to 0.244) |
| **Maldives** | 424.47 (329.40-552.89) | 601.49 (471.53-786.40) | 1,870.91 (1,496.63-2,408.53) | 619.06 (486.08-803.07) | 0.03 (-0.02 to 0.08) | 0.082 (0.073 to 0.091) |
| **Mongolia** | 4,854.18 (3,717.89-6,265.54) | 507.69 (387.87-661.82) | 10,600.52 (8,189.32-13,896.88) | 544.58 (413.48-704.44) | 0.07 (0.02 to 0.13) | 0.221 (0.212 to 0.231) |
| **Myanmar** | 118,340.52 (93,008.70-156,133.07) | 613.93 (479.10-797.70) | 267,795.42 (211,908.70-352,205.79) | 614.44 (480.43-795.77) | 0 (-0.04 to 0.05) | 0.005 (-0.004 to 0.013) |
| **Nepal** | 34,653.70 (26,729.16-45,519.31) | 471.77 (362.87-619.63) | 96,213.46 (73,202.62-125,249.60) | 471.74 (359.90-618.89) | 0 (-0.04 to 0.04) | -0.002 (-0.017 to 0.012) |
| **Oman** | 1,615.23 (1,247.98-2,101.23) | 292.89 (225.07-380.76) | 5,827.22 (4,557.16-7,636.55) | 365.26 (276.39-482.95) | 0.25 (0.18 to 0.3) | 0.698 (0.683 to 0.713) |
| **Pakistan** | 270,729.01 (206,527.92-355,785.16) | 554.73 (429.37-728.70) | 548,173.57 (427,140.93-726,636.82) | 558.61 (429.50-735.69) | 0.01 (-0.03 to 0.04) | 0.025 (0.017 to 0.032) |
| **Palestine** | 2,436.60 (1,858.79-3,179.30) | 323.20 (244.98-422.73) | 7,196.66 (5,524.61-9,404.90) | 347.45 (265.23-453.98) | 0.08 (0.03 to 0.12) | 0.237 (0.228 to 0.247) |
| **Philippines** | 160,947.18 (126,927.88-211,314.06) | 648.69 (510.61-837.57) | 466,916.29 (369,506.77-610,795.27) | 653.73 (513.13-843.12) | 0.01 (0 to 0.02) | 0.028 (0.022 to 0.034) |
| **Qatar** | 298.06 (228.10-388.99) | 363.49 (278.22-475.53) | 3,004.96 (2,251.21-3,953.06) | 402.59 (305.76-529.29) | 0.11 (0.06 to 0.16) | 0.330 (0.303 to 0.357) |
| **Republic of Korea** | 167,647.19 (132,028.04-217,480.24) | 610.53 (486.70-785.88) | 628,474.25 (544,532.76-739,494.39) | 675.14 (584.70-790.59) | 0.11 (-0.04 to 0.28) | 0.470 (0.360 to 0.580) |
| **Saudi Arabia** | 15,737.44 (12,130.38-20,449.75) | 330.72 (250.33-435.24) | 54,167.00 (42,124.93-71,479.10) | 391.86 (301.89-511.74) | 0.18 (0.14 to 0.25) | 0.548 (0.534 to 0.562) |
| **Singapore** | 10,360.83 (8,189.62-13,361.26) | 496.12 (394.24-643.05) | 36,795.45 (28,982.34-47,619.92) | 430.26 (339.42-557.39) | -0.13 (-0.17 to -0.1) | -0.442 (-0.472 to -0.413) |
| **Sri Lanka** | 55,985.87 (44,215.54-73,509.66) | 600.31 (472.18-777.41) | 163,229.15 (128,662.14-213,321.89) | 632.17 (494.65-817.05) | 0.05 (0.01 to 0.1) | 0.165 (0.156 to 0.175) |
| **Syrian Arab Republic** | 15,094.73 (11,604.77-19,661.37) | 348.36 (264.02-453.87) | 41,606.02 (32,219.89-54,570.11) | 377.81 (288.12-497.32) | 0.08 (0.04 to 0.13) | 0.255 (0.246 to 0.265) |
| **Tajikistan** | 11,955.47 (9,330.95-15,430.53) | 476.78 (369.22-619.07) | 23,849.65 (18,509.22-31,220.50) | 489.48 (373.73-641.48) | 0.03 (-0.02 to 0.08) | 0.101 (0.086 to 0.115) |
| **Thailand** | 176,404.59 (139,407.65-229,043.44) | 577.24 (454.35-745.36) | 661,374.37 (516,881.41-864,805.43) | 603.80 (470.51-784.54) | 0.05 (0 to 0.09) | 0.155 (0.140 to 0.170) |
| **Timor-Leste** | 1,280.06 (1,022.70-1,637.79) | 585.93 (462.52-761.76) | 4,679.77 (3,684.69-6,091.90) | 605.83 (476.38-787.01) | 0.03 (-0.02 to 0.09) | 0.112 (0.104 to 0.120) |
| **Türkiye** | 99,437.22 (77,990.81-127,593.73) | 332.71 (262.14-425.52) | 247,837.12 (221,168.36-275,261.69) | 282.89 (252.21-314.68) | -0.15 (-0.31 to 0.04) | -0.515 (-0.584 to -0.447) |
| **Turkmenistan** | 8,443.59 (6,459.92-11,054.11) | 501.72 (380.44-654.49) | 19,896.45 (15,619.26-25,582.99) | 563.75 (432.79-729.21) | 0.12 (0.07 to 0.19) | 0.382 (0.376 to 0.388) |
| **United Arab Emirates** | 1,293.67 (989.60-1,673.84) | 381.72 (289.37-494.96) | 13,474.73 (9,821.02-18,266.30) | 424.69 (326.48-554.82) | 0.11 (0.06 to 0.17) | 0.359 (0.345 to 0.372) |
| **Uzbekistan** | 49,809.29 (38,683.10-65,010.75) | 460.42 (354.54-596.53) | 112,674.87 (87,823.78-148,398.36) | 496.56 (380.60-651.61) | 0.08 (0.02 to 0.14) | 0.244 (0.229 to 0.259) |
| **Viet Nam** | 214,245.36 (169,209.19-280,475.71) | 581.97 (454.92-753.15) | 581,495.83 (457,784.43-771,131.22) | 644.90 (502.66-844.07) | 0.11 (0.06 to 0.16) | 0.333 (0.315 to 0.351) |
| **Yemen** | 11,693.01 (8,973.81-15,102.03) | 307.38 (234.12-401.08) | 38,432.05 (29,592.50-49,670.58) | 337.23 (255.75-443.55) | 0.1 (0.05 to 0.16) | 0.299 (0.289 to 0.309) |

Supplementary Table S3: Incidence of AF/AFL in Asia in 1990 and 2021.

|  | **Counts (95% UI, 1990)** | **ASIR 100,000**  **(95% UI, 1990)** | **Counts**  **(95% UI, 2021)** | **ASIR 100,000**  **(95% UI, 2021)** | **PC, (95% UI, 1990-2021)** | **AAPC (95% CI)** |
| --- | --- | --- | --- | --- | --- | --- |
| **Afghanistan** | 1,905.71 (1,414.58-2,570.98) | 31.95 (23.70-42.95) | 2,735.22 (2,055.65-3,602.50) | 33.33 (24.48-44.48) | 0.04 (0 to 0.09) | 0.133 (0.118 to 0.148) |
| **Armenia** | 1,139.96 (871.94-1,501.74) | 43.01 (32.14-56.20) | 2,012.80 (1,478.80-2,662.39) | 45.65 (34.23-60.63) | 0.06 (0.01 to 0.11) | 0.204 (0.186 to 0.222) |
| **Azerbaijan** | 2,078.59 (1,577.26-2,727.18) | 43.33 (32.63-56.59) | 4,617.29 (3,522.60-6,113.31) | 44.90 (33.78-58.97) | 0.04 (-0.01 to 0.09) | 0.125 (0.115 to 0.134) |
| **Bahrain** | 53.39 (40.77-70.24) | 35.35 (26.17-47.72) | 296.98 (229.04-382.34) | 36.29 (26.61-48.17) | 0.03 (-0.03 to 0.07) | 0.083 (0.069 to 0.097) |
| **Bangladesh** | 19,440.25 (14,830.21-25,449.36) | 45.54 (33.71-59.79) | 60,981.74 (45,613.84-80,831.08) | 46.19 (34.26-61.31) | 0.01 (-0.04 to 0.06) | 0.071 (0.040 to 0.102) |
| **Bhutan** | 93.78 (70.75-122.97) | 45.60 (33.80-60.36) | 269.05 (202.21-360.16) | 46.27 (34.64-61.83) | 0.01 (-0.03 to 0.06) | 0.047 (0.032 to 0.062) |
| **Brunei Darussalam** | 48.25 (37.87-61.14) | 43.19 (33.56-56.47) | 129.79 (100.90-165.95) | 35.52 (27.30-45.61) | -0.18 (-0.22 to -0.14) | -0.603 (-0.622 to -0.584) |
| **Cambodia** | 2,095.88 (1,615.45-2,726.97) | 53.26 (40.70-70.29) | 5,972.85 (4,646.62-7,811.59) | 52.94 (40.15-69.72) | -0.01 (-0.04 to 0.03) | -0.027 (-0.037 to -0.018) |
| **China** | 306,585.30 (234,243.07-404,868.18) | 42.63 (32.40-56.46) | 916,180.08 (707,383.77-1,201,380.79) | 44.92 (34.96-59.42) | 0.05 (0.01 to 0.09) | 0.192 (0.119 to 0.264) |
| **Cyprus** | 476.62 (346.93-624.84) | 55.82 (42.00-72.70) | 928.48 (719.89-1,217.88) | 42.95 (34.02-55.26) | -0.23 (-0.31 to -0.14) | -0.903 (-0.982 to -0.824) |
| **Democratic People's Republic of Korea** | 7,060.14 (5,521.80-9,164.81) | 49.69 (38.11-65.61) | 15,152.45 (11,772.24-20,143.61) | 48.09 (37.23-63.29) | -0.03 (-0.07 to 0.01) | -0.104 (-0.119 to -0.088) |
| **Georgia** | 2,950.38 (2,186.73-3,909.55) | 47.31 (35.38-61.93) | 2,829.69 (2,114.35-3,689.65) | 47.21 (35.92-61.51) | 0 (-0.04 to 0.04) | 0.004 (-0.014 to 0.021) |
| **India** | 198,418.84 (151,419.86-263,224.23) | 50.27 (37.44-66.79) | 570,120.90 (430,165.09-762,853.23) | 51.45 (38.36-68.54) | 0.02 (0.01 to 0.03) | 0.078 (0.056 to 0.099) |
| **Indonesia** | 55,881.45 (43,452.66-72,892.02) | 64.43 (49.08-85.90) | 145,423.01 (112,648.14-190,123.43) | 66.36 (50.84-87.95) | 0.03 (0.01 to 0.05) | 0.099 (0.093 to 0.104) |
| **Iran (Islamic Republic of)** | 8,136.43 (6,131.54-10,786.79) | 37.34 (27.62-50.20) | 29,418.30 (22,665.13-38,723.09) | 40.56 (30.00-54.38) | 0.09 (0.07 to 0.1) | 0.246 (0.228 to 0.265) |
| **Iraq** | 2,716.94 (2,025.29-3,569.63) | 36.58 (26.52-48.72) | 8,150.81 (6,210.28-10,702.55) | 38.36 (28.13-51.24) | 0.05 (-0.01 to 0.11) | 0.152 (0.145 to 0.159) |
| **Israel** | 3,550.82 (2,613.92-4,680.23) | 70.89 (53.65-92.18) | 11,109.65 (8,994.87-12,779.09) | 91.98 (75.23-105.16) | 0.3 (0.08 to 0.63) | 0.934 (0.888 to 0.979) |
| **Japan** | 70,805.37 (54,524.97-93,790.49) | 42.03 (32.59-55.25) | 107,285.28 (77,974.54-144,806.52) | 31.86 (24.84-41.91) | -0.24 (-0.26 to -0.22) | -0.714 (-0.846 to -0.582) |
| **Jordan** | 385.70 (295.04-505.09) | 34.36 (25.04-46.19) | 2,471.59 (1,877.97-3,282.47) | 37.00 (27.48-49.67) | 0.08 (0.03 to 0.13) | 0.233 (0.224 to 0.242) |
| **Kazakhstan** | 5,935.25 (4,511.21-7,796.48) | 48.19 (36.41-63.55) | 8,828.50 (6,625.85-11,518.66) | 48.85 (37.09-63.65) | 0.01 (-0.03 to 0.06) | 0.035 (0.015 to 0.055) |
| **Kuwait** | 188.09 (147.41-241.50) | 35.46 (25.71-47.38) | 1,013.24 (804.18-1,301.49) | 37.87 (27.94-50.89) | 0.07 (0.01 to 0.12) | 0.195 (0.176 to 0.214) |
| **Kyrgyzstan** | 1,202.66 (908.72-1,594.54) | 41.46 (31.00-54.33) | 1,957.11 (1,491.35-2,571.82) | 41.13 (31.43-53.83) | -0.01 (-0.05 to 0.04) | -0.030 (-0.055 to -0.005) |
| **Lao People's Democratic Republic** | 1,000.02 (756.57-1,323.94) | 55.76 (42.56-74.12) | 2,332.85 (1,815.70-3,027.08) | 54.97 (41.92-72.84) | -0.01 (-0.05 to 0.03) | -0.032 (-0.039 to -0.024) |
| **Lebanon** | 649.52 (493.99-854.85) | 33.33 (24.80-44.87) | 2,243.58 (1,665.49-3,000.94) | 36.25 (26.97-48.31) | 0.09 (0.03 to 0.14) | 0.271 (0.260 to 0.282) |
| **Malaysia** | 5,040.96 (3,955.20-6,559.15) | 57.12 (43.61-75.30) | 15,961.55 (12,194.39-20,997.95) | 58.73 (44.38-78.66) | 0.03 (-0.02 to 0.07) | 0.096 (0.077 to 0.114) |
| **Maldives** | 41.94 (32.36-54.85) | 53.66 (40.67-71.29) | 181.46 (145.41-232.58) | 54.10 (41.34-71.72) | 0.01 (-0.04 to 0.06) | 0.024 (0.015 to 0.034) |
| **Mongolia** | 449.98 (338.20-592.74) | 44.09 (33.03-57.99) | 1,033.99 (782.46-1,341.25) | 45.64 (34.27-60.09) | 0.04 (-0.01 to 0.09) | 0.112 (0.102 to 0.122) |
| **Myanmar** | 11,699.93 (8,929.99-15,322.13) | 57.50 (43.40-76.58) | 25,401.09 (19,547.76-33,339.47) | 55.75 (42.05-74.46) | -0.03 (-0.07 to 0.01) | -0.095 (-0.105 to -0.085) |
| **Nepal** | 3,700.30 (2,804.51-4,891.18) | 45.84 (34.18-61.00) | 9,674.61 (7,231.82-12,861.98) | 44.58 (33.43-59.27) | -0.03 (-0.07 to 0.01) | -0.088 (-0.105 to -0.072) |
| **Oman** | 176.28 (134.34-226.54) | 28.99 (21.37-38.68) | 643.44 (503.59-828.86) | 34.43 (25.27-46.32) | 0.19 (0.13 to 0.25) | 0.567 (0.550 to 0.584) |
| **Pakistan** | 27,496.91 (20,886.88-36,777.32) | 53.26 (39.62-71.06) | 57,873.94 (44,288.70-75,860.60) | 53.85 (40.30-71.60) | 0.01 (-0.02 to 0.05) | 0.031 (0.021 to 0.040) |
| **Palestine** | 252.32 (188.68-337.66) | 31.95 (23.55-42.92) | 748.62 (570.37-981.62) | 33.18 (24.40-44.66) | 0.04 (0 to 0.09) | 0.120 (0.112 to 0.127) |
| **Philippines** | 15,964.31 (12,389.76-20,940.74) | 59.63 (45.31-79.40) | 45,632.13 (35,428.48-59,597.99) | 60.30 (45.81-80.23) | 0.01 (0 to 0.02) | 0.038 (0.031 to 0.045) |
| **Qatar** | 36.33 (28.56-46.09) | 35.20 (25.60-46.99) | 365.73 (282.57-474.27) | 37.60 (27.56-50.65) | 0.07 (0.01 to 0.12) | 0.200 (0.175 to 0.224) |
| **Republic of Korea** | 14,766.02 (11,525.39-18,923.78) | 49.39 (38.37-64.76) | 42,194.07 (34,816.66-52,133.40) | 47.21 (39.34-57.49) | -0.04 (-0.16 to 0.09) | -0.002 (-0.127 to 0.124) |
| **Saudi Arabia** | 1,685.53 (1,283.81-2,222.85) | 32.66 (24.17-44.12) | 6,095.80 (4,757.13-7,921.38) | 36.92 (27.20-49.85) | 0.13 (0.08 to 0.19) | 0.397 (0.381 to 0.413) |
| **Singapore** | 898.69 (703.56-1,153.87) | 39.69 (30.61-52.17) | 2,861.48 (2,242.47-3,725.86) | 33.07 (25.69-42.97) | -0.17 (-0.2 to -0.13) | -0.567 (-0.595 to -0.538) |
| **Sri Lanka** | 5,376.86 (4,144.55-7,100.48) | 54.08 (41.22-72.82) | 14,672.23 (11,154.36-19,567.37) | 55.75 (42.39-73.31) | 0.03 (-0.01 to 0.09) | 0.098 (0.085 to 0.111) |
| **Syrian Arab Republic** | 1,567.33 (1,191.35-2,036.61) | 34.12 (25.03-45.49) | 4,228.06 (3,162.89-5,673.72) | 35.28 (25.74-47.13) | 0.03 (-0.01 to 0.08) | 0.106 (0.094 to 0.118) |
| **Tajikistan** | 1,084.19 (831.61-1,420.11) | 41.09 (31.09-53.75) | 2,332.88 (1,786.16-3,072.30) | 40.85 (30.53-53.30) | -0.01 (-0.05 to 0.04) | 0.001 (-0.018 to 0.019) |
| **Thailand** | 17,124.16 (13,433.12-22,526.63) | 52.67 (40.36-69.23) | 57,917.85 (44,291.55-77,052.24) | 53.17 (40.48-70.77) | 0.01 (-0.04 to 0.06) | 0.036 (0.012 to 0.059) |
| **Timor-Leste** | 133.58 (105.82-169.60) | 54.47 (41.71-72.09) | 439.27 (332.05-585.67) | 54.61 (41.79-72.26) | 0 (-0.04 to 0.05) | 0.019 (0.012 to 0.025) |
| **Türkiye** | 10,042.30 (7,801.56-12,979.26) | 32.02 (24.36-41.70) | 24,773.75 (21,786.11-28,278.44) | 27.35 (24.06-31.31) | -0.15 (-0.31 to 0.04) | -0.522 (-0.567 to -0.476) |
| **Turkmenistan** | 793.83 (597.37-1,038.73) | 43.24 (32.26-56.89) | 1,872.47 (1,439.13-2,433.13) | 47.04 (35.47-60.99) | 0.09 (0.04 to 0.15) | 0.282 (0.272 to 0.292) |
| **United Arab Emirates** | 155.15 (120.11-198.64) | 37.02 (27.30-49.36) | 1,662.05 (1,236.05-2,172.89) | 39.65 (29.19-52.80) | 0.07 (0.02 to 0.13) | 0.231 (0.210 to 0.252) |
| **Uzbekistan** | 4,414.47 (3,384.03-5,822.01) | 39.64 (29.94-52.67) | 10,746.09 (8,147.55-14,123.80) | 41.51 (30.94-54.26) | 0.05 (-0.01 to 0.11) | 0.151 (0.127 to 0.175) |
| **Viet Nam** | 20,315.10 (15,605.11-26,791.42) | 53.94 (41.15-71.13) | 53,982.88 (41,974.07-71,154.44) | 57.26 (43.75-76.67) | 0.06 (0.01 to 0.11) | 0.189 (0.171 to 0.207) |
| **Yemen** | 1,282.21 (964.00-1,685.14) | 31.04 (22.91-41.47) | 4,104.83 (3,060.81-5,426.72) | 32.90 (24.21-44.14) | 0.06 (0.01 to 0.12) | 0.187 (0.176 to 0.198) |

Supplementary Table S4: Death of AF/AFL in Asia in 1990 and 2021.

|  | **Counts (95% UI, 1990)** | **ASMR 100,000**  **(95% UI, 1990)** | **Counts**  **(95% UI, 2021)** | **ASMR 100,000**  **(95% UI, 2021)** | **PC, (95% UI, 1990-2021)** | **AAPC (95% CI)** |
| --- | --- | --- | --- | --- | --- | --- |
| **Afghanistan** | 112.59 (59.39-189.72) | 2.75 (1.46-4.55) | 179.27 (110.91-263.87) | 3.28 (2.04-4.86) | 0.19 (-0.07 to 0.61) | 0.504 (0.443 to 0.566) |
| **Armenia** | 30.86 (26.77-35.45) | 1.52 (1.31-1.77) | 107.96 (93.39-121.77) | 2.53 (2.18-2.86) | 0.67 (0.32 to 1.01) | 1.158 (0.446 to 1.870) |
| **Azerbaijan** | 58.56 (47.51-76.51) | 1.65 (1.34-2.14) | 123.57 (92.95-154.47) | 1.79 (1.39-2.21) | 0.08 (-0.22 to 0.45) | 0.238 (-0.015 to 0.492) |
| **Bahrain** | 3.67 (1.90-4.84) | 6.97 (3.40-9.27) | 14.66 (8.06-19.91) | 5.66 (2.58-7.90) | -0.19 (-0.39 to -0.01) | -0.942 (-1.369 to -0.515) |
| **Bangladesh** | 1,026.32 (566.68-1,541.92) | 3.26 (1.75-4.90) | 4,551.85 (3,251.65-6,400.31) | 5.00 (3.64-6.99) | 0.53 (0.14 to 1.36) | 1.693 (1.165 to 2.220) |
| **Bhutan** | 3.23 (1.74-5.12) | 2.64 (1.42-4.22) | 21.68 (14.99-30.15) | 4.47 (3.09-6.28) | 0.69 (0.19 to 1.48) | 1.699 (1.629 to 1.770) |
| **Brunei Darussalam** | 5.00 (3.68-6.40) | 7.15 (5.28-9.27) | 11.56 (9.64-13.49) | 6.22 (5.18-7.43) | -0.13 (-0.32 to 0.19) | -0.395 (-0.751 to -0.039) |
| **Cambodia** | 74.35 (55.34-108.51) | 3.19 (2.31-4.70) | 278.53 (216.56-359.13) | 4.30 (3.27-5.61) | 0.35 (0.01 to 0.76) | 0.992 (0.957 to 1.028) |
| **China** | 16,448.97 (13,239.72-20,520.66) | 4.93 (3.88-6.17) | 64,728.22 (51,764.98-77,729.27) | 4.33 (3.43-5.23) | -0.12 (-0.35 to 0.16) | -0.441 (-0.695 to -0.188) |
| **Cyprus** | 58.81 (40.39-77.94) | 14.11 (9.57-19.11) | 123.39 (102.38-143.27) | 8.36 (6.79-9.85) | -0.41 (-0.56 to -0.13) | -2.012 (-2.506 to -1.517) |
| **Democratic People's Republic of Korea** | 420.75 (296.01-577.80) | 5.07 (3.43-7.31) | 1,167.33 (885.59-1,665.41) | 5.09 (3.77-7.73) | 0 (-0.24 to 0.37) | -0.006 (-0.080 to 0.068) |
| **Georgia** | 167.88 (125.54-202.39) | 3.26 (2.37-3.99) | 340.62 (292.56-380.42) | 5.05 (4.37-5.62) | 0.55 (0.21 to 1.16) | 2.364 (0.991 to 3.737) |
| **India** | 5,413.26 (3,290.59-8,479.79) | 2.15 (1.32-3.41) | 27,887.86 (20,425.59-34,928.45) | 3.44 (2.50-4.32) | 0.6 (0.15 to 1.18) | 1.719 (1.318 to 2.119) |
| **Indonesia** | 1,899.26 (1,376.43-2,467.76) | 3.60 (2.54-4.93) | 6,987.38 (5,444.54-8,420.82) | 6.36 (4.84-7.80) | 0.77 (0.26 to 1.35) | 1.820 (1.779 to 1.861) |
| **Iran (Islamic Republic of)** | 410.65 (305.94-509.91) | 3.32 (2.42-4.15) | 1,948.93 (1,462.62-2,233.11) | 3.31 (2.47-3.80) | 0 (-0.22 to 0.28) | -0.047 (-0.154 to 0.061) |
| **Iraq** | 226.14 (161.23-304.29) | 3.25 (2.31-4.38) | 647.08 (481.16-825.67) | 4.85 (3.51-6.19) | 0.49 (0.06 to 1.32) | 1.398 (1.210 to 1.587) |
| **Israel** | 256.14 (227.66-272.81) | 6.31 (5.50-6.76) | 714.14 (580.09-793.63) | 4.84 (3.98-5.35) | -0.23 (-0.3 to -0.18) | -0.991 (-1.239 to -0.743) |
| **Japan** | 4,428.62 (3,882.85-4,699.36) | 3.04 (2.63-3.25) | 13,836.71 (10,679.10-15,687.21) | 2.33 (1.86-2.58) | -0.23 (-0.29 to -0.2) | -0.865 (-1.053 to -0.677) |
| **Jordan** | 23.34 (19.14-28.34) | 3.14 (2.54-3.78) | 102.51 (80.47-125.37) | 2.72 (2.12-3.29) | -0.13 (-0.36 to 0.1) | -0.599 (-0.931 to -0.268) |
| **Kazakhstan** | 212.68 (172.31-265.45) | 2.20 (1.75-2.79) | 349.80 (305.15-395.25) | 2.97 (2.57-3.34) | 0.35 (0.03 to 0.73) | 1.020 (0.579 to 1.461) |
| **Kuwait** | 8.55 (7.07-9.64) | 2.59 (2.08-2.95) | 60.33 (48.11-71.77) | 3.00 (2.40-3.57) | 0.16 (-0.01 to 0.33) | 1.668 (0.661 to 2.674) |
| **Kyrgyzstan** | 45.90 (38.11-56.22) | 2.01 (1.65-2.47) | 72.56 (61.07-83.73) | 2.17 (1.82-2.49) | 0.08 (-0.13 to 0.33) | -0.010 (-0.651 to 0.632) |
| **Lao People's Democratic Republic** | 40.81 (27.31-60.09) | 4.19 (2.82-6.25) | 129.57 (101.63-169.19) | 5.14 (4.04-6.72) | 0.23 (-0.09 to 0.74) | 0.635 (0.580 to 0.690) |
| **Lebanon** | 72.72 (40.94-113.32) | 4.96 (2.67-7.76) | 238.88 (192.70-307.78) | 3.41 (2.75-4.35) | -0.31 (-0.58 to 0.33) | -1.111 (-1.267 to -0.955) |
| **Malaysia** | 236.80 (193.23-289.39) | 3.22 (2.60-3.94) | 930.75 (785.66-1,069.44) | 4.94 (4.12-5.74) | 0.54 (0.23 to 1) | 1.216 (0.949 to 1.484) |
| **Maldives** | 1.40 (0.93-1.94) | 4.05 (2.57-5.55) | 9.62 (7.17-11.94) | 4.06 (2.99-5.05) | 0 (-0.34 to 0.55) | 0.041 (-0.104 to 0.185) |
| **Mongolia** | 15.02 (11.57-19.40) | 2.00 (1.54-2.61) | 26.02 (20.30-32.81) | 1.91 (1.49-2.44) | -0.05 (-0.28 to 0.25) | -0.354 (-0.623 to -0.084) |
| **Myanmar** | 503.97 (349.51-721.92) | 4.16 (2.93-5.94) | 1,630.45 (1,236.34-2,083.85) | 5.15 (3.90-6.60) | 0.24 (-0.13 to 0.72) | 0.703 (0.632 to 0.774) |
| **Nepal** | 116.10 (63.42-184.47) | 2.31 (1.25-3.65) | 588.54 (400.96-828.98) | 4.05 (2.76-5.80) | 0.75 (0.26 to 1.6) | 1.889 (1.795 to 1.983) |
| **Oman** | 16.60 (11.97-22.98) | 4.03 (2.91-5.59) | 45.33 (35.83-55.15) | 4.73 (3.62-5.93) | 0.17 (-0.23 to 0.82) | 0.377 (-0.116 to 0.871) |
| **Pakistan** | 1,035.21 (659.69-1,504.30) | 2.80 (1.81-4.06) | 3,114.82 (2,310.54-4,332.20) | 4.58 (3.33-6.38) | 0.64 (0.22 to 1.26) | 1.503 (1.429 to 1.576) |
| **Palestine** | 28.50 (20.98-37.10) | 5.11 (3.74-6.68) | 68.14 (55.16-81.97) | 5.37 (4.31-6.66) | 0.05 (-0.2 to 0.43) | 0.159 (-0.044 to 0.362) |
| **Philippines** | 690.84 (594.52-803.10) | 5.26 (4.52-6.12) | 2,445.05 (2,054.33-2,911.68) | 4.82 (4.02-5.81) | -0.08 (-0.25 to 0.07) | -0.127 (-0.353 to 0.099) |
| **Qatar** | 2.32 (1.11-3.09) | 7.37 (3.17-10.02) | 9.99 (5.41-14.98) | 4.06 (1.45-6.04) | -0.45 (-0.62 to -0.27) | -2.032 (-2.991 to -1.073) |
| **Republic of Korea** | 553.03 (422.02-873.10) | 3.38 (2.55-5.22) | 3,164.62 (2,356.33-3,784.62) | 3.50 (2.60-4.17) | 0.04 (-0.5 to 0.46) | 0.225 (0.036 to 0.414) |
| **Saudi Arabia** | 102.36 (78.20-141.98) | 2.97 (2.27-4.12) | 248.87 (197.43-305.44) | 3.35 (2.67-4.11) | 0.13 (-0.22 to 0.54) | 0.320 (0.213 to 0.427) |
| **Singapore** | 31.60 (29.50-33.05) | 1.94 (1.78-2.04) | 99.20 (83.89-108.26) | 1.22 (1.03-1.33) | -0.37 (-0.43 to -0.32) | -1.594 (-1.703 to -1.485) |
| **Sri Lanka** | 208.68 (179.90-245.63) | 3.64 (3.07-4.33) | 777.43 (556.22-1,029.12) | 4.05 (2.89-5.35) | 0.11 (-0.24 to 0.54) | 0.547 (0.143 to 0.951) |
| **Syrian Arab Republic** | 152.53 (108.96-200.63) | 4.26 (3.02-5.62) | 318.30 (209.60-419.40) | 4.57 (2.89-5.92) | 0.07 (-0.28 to 0.59) | 0.190 (-0.350 to 0.730) |
| **Tajikistan** | 27.58 (20.03-40.71) | 1.26 (0.90-1.90) | 39.23 (31.80-48.42) | 1.15 (0.92-1.44) | -0.09 (-0.4 to 0.28) | -0.397 (-0.791 to -0.003) |
| **Thailand** | 1,205.89 (930.26-1,571.87) | 5.81 (4.39-7.57) | 5,063.97 (3,744.44-6,439.68) | 4.56 (3.39-5.83) | -0.21 (-0.44 to 0.1) | -0.743 (-0.951 to -0.535) |
| **Timor-Leste** | 4.89 (3.13-6.97) | 3.56 (2.28-5.09) | 22.15 (16.90-28.45) | 4.54 (3.47-5.80) | 0.28 (-0.11 to 0.94) | 0.819 (0.773 to 0.865) |
| **Türkiye** | 835.65 (658.65-1,099.76) | 3.59 (2.79-4.75) | 2,923.39 (2,332.01-3,533.34) | 3.88 (3.05-4.69) | 0.08 (-0.22 to 0.46) | 0.609 (0.000 to 1.218) |
| **Turkmenistan** | 28.70 (24.44-34.02) | 2.19 (1.83-2.65) | 86.31 (67.71-111.69) | 2.89 (2.27-3.76) | 0.32 (0.01 to 0.68) | 0.917 (0.636 to 1.199) |
| **United Arab Emirates** | 6.77 (4.80-9.69) | 3.62 (2.64-5.17) | 30.13 (23.51-37.41) | 3.82 (2.51-4.93) | 0.06 (-0.3 to 0.47) | 0.542 (-0.782 to 1.866) |
| **Uzbekistan** | 97.03 (57.37-157.52) | 1.03 (0.60-1.69) | 240.33 (203.62-277.60) | 1.39 (1.18-1.60) | 0.36 (-0.22 to 1.38) | 0.926 (0.392 to 1.459) |
| **Viet Nam** | 1,219.11 (961.39-1,630.29) | 4.14 (3.22-5.56) | 4,014.82 (2,990.38-5,030.49) | 5.83 (4.35-7.36) | 0.41 (0.05 to 0.87) | 1.098 (1.046 to 1.150) |
| **Yemen** | 75.52 (44.41-121.46) | 3.08 (1.79-4.86) | 290.81 (198.18-403.56) | 3.74 (2.57-5.12) | 0.22 (-0.17 to 0.84) | 0.614 (0.516 to 0.713) |

Supplementary Table S5: DALYs for AF/AFL in Asia in 1990 and 2021.

|  | **Counts (95% UI)** | **ASR DALYs 100,000**  **(95% UI, 1990)** | **Counts (95% UI, 2021)** | **ASR DALYs 100,000**  **(95% UI, 2021)** | **PC, (95% UI, 1990-2021)** | **AAPC (95% CI)** |
| --- | --- | --- | --- | --- | --- | --- |
| **Afghanistan** | 3,394.12 (2,188.10-4,976.67) | 63.05 (40.48-92.63) | 5,074.27 (3,663.27-6,760.92) | 72.08 (51.55-95.15) | 0.14 (-0.06 to 0.37) | 0.408 (0.370 to 0.445) |
| **Armenia** | 1,450.82 (1,073.85-1,905.47) | 60.37 (45.72-79.07) | 3,354.89 (2,606.74-4,231.36) | 76.87 (59.76-96.05) | 0.27 (0.16 to 0.41) | 0.991 (0.475 to 1.507) |
| **Azerbaijan** | 2,729.44 (1,993.84-3,648.22) | 62.51 (46.28-83.71) | 5,901.94 (4,360.70-7,950.72) | 67.92 (51.38-90.14) | 0.09 (-0.03 to 0.23) | 0.235 (0.157 to 0.313) |
| **Bahrain** | 101.33 (65.33-127.54) | 113.06 (66.97-143.09) | 442.01 (315.55-569.28) | 94.91 (57.64-122.79) | -0.16 (-0.33 to -0.02) | -0.860 (-1.144 to -0.576) |
| **Bangladesh** | 29,953.52 (20,406.14-40,048.87) | 78.66 (52.88-105.79) | 109,145.91 (82,746.99-147,678.87) | 97.30 (74.50-129.97) | 0.24 (0.03 to 0.52) | 0.853 (0.563 to 1.142) |
| **Bhutan** | 122.67 (85.69-166.87) | 71.66 (49.36-97.91) | 502.92 (378.64-648.76) | 93.85 (70.80-121.02) | 0.31 (0.07 to 0.59) | 0.855 (0.809 to 0.901) |
| **Brunei Darussalam** | 124.29 (102.23-149.04) | 144.66 (117.27-173.57) | 327.98 (271.01-388.32) | 122.03 (102.46-143.98) | -0.16 (-0.29 to 0.03) | -0.528 (-0.734 to -0.322) |
| **Cambodia** | 2,927.15 (2,222.57-3,785.07) | 86.33 (66.07-113.06) | 9,138.00 (7,031.03-11,349.11) | 98.48 (76.53-122.26) | 0.14 (-0.03 to 0.33) | 0.433 (0.399 to 0.468) |
| **China** | 508,610.26 (395,853.05-638,618.04) | 93.28 (75.14-115.50) | 1,653,116.54 (1,303,681.18-2,056,459.47) | 89.76 (72.13-109.67) | -0.04 (-0.19 to 0.14) | -0.173 (-0.307 to -0.039) |
| **Cyprus** | 1,241.49 (932.15-1,549.76) | 217.88 (160.27-274.78) | 2,332.61 (1,967.18-2,795.32) | 129.79 (108.76-154.10) | -0.4 (-0.53 to -0.2) | -1.669 (-1.931 to -1.408) |
| **Democratic People's Republic of Korea** | 12,100.72 (9,158.51-15,413.76) | 103.92 (79.23-132.66) | 29,339.95 (22,444.44-38,028.85) | 103.98 (80.31-138.02) | 0 (-0.14 to 0.19) | -0.007 (-0.046 to 0.031) |
| **Georgia** | 5,208.88 (3,806.85-6,549.93) | 89.40 (64.79-113.19) | 7,760.16 (6,544.61-9,213.29) | 122.39 (102.90-145.33) | 0.37 (0.2 to 0.66) | 1.195 (0.312 to 2.077) |
| **India** | 239,570.28 (175,321.87-323,221.40) | 69.36 (51.38-93.59) | 855,144.53 (645,560.42-1,094,553.83) | 86.05 (65.71-108.52) | 0.24 (0.06 to 0.44) | 0.740 (0.593 to 0.887) |
| **Indonesia** | 75,340.09 (58,204.18-96,155.24) | 101.59 (77.89-129.42) | 223,354.32 (178,283.84-274,231.74) | 133.13 (106.77-160.61) | 0.31 (0.11 to 0.53) | 0.851 (0.836 to 0.867) |
| **Iran (Islamic Republic of)** | 12,115.72 (9,430.07-15,214.42) | 70.54 (55.40-88.33) | 47,524.67 (37,527.75-58,270.85) | 72.39 (57.36-88.32) | 0.03 (-0.13 to 0.18) | 0.034 (-0.032 to 0.101) |
| **Iraq** | 5,572.19 (4,347.65-6,977.87) | 76.45 (59.52-96.57) | 16,445.31 (13,020.41-19,937.68) | 93.63 (74.24-114.00) | 0.22 (-0.02 to 0.66) | 0.732 (0.618 to 0.846) |
| **Israel** | 6,748.80 (5,501.45-8,272.23) | 145.13 (119.45-175.94) | 19,690.96 (15,653.54-24,213.96) | 148.81 (117.35-184.36) | 0.03 (-0.07 to 0.15) | -0.006 (-0.299 to 0.288) |
| **Japan** | 132,019.54 (106,095.83-163,379.34) | 81.38 (66.07-100.34) | 270,640.59 (220,302.12-330,701.80) | 61.78 (50.37-76.42) | -0.24 (-0.27 to -0.22) | -0.795 (-0.954 to -0.637) |
| **Jordan** | 658.27 (525.74-813.42) | 68.66 (55.41-84.59) | 3,377.04 (2,602.29-4,222.85) | 63.06 (49.36-78.25) | -0.08 (-0.24 to 0.06) | -0.383 (-0.556 to -0.211) |
| **Kazakhstan** | 8,359.20 (6,441.87-11,000.39) | 74.49 (57.90-97.30) | 12,990.38 (10,082.20-16,722.83) | 85.35 (68.14-108.69) | 0.15 (0.02 to 0.3) | 0.332 (0.092 to 0.572) |
| **Kuwait** | 272.05 (216.51-338.64) | 62.26 (49.55-76.91) | 1,524.69 (1,212.46-1,888.98) | 66.57 (52.84-82.17) | 0.07 (-0.03 to 0.17) | 0.713 (0.194 to 1.232) |
| **Kyrgyzstan** | 1,739.53 (1,344.28-2,258.21) | 64.88 (50.53-83.45) | 2,816.27 (2,186.80-3,621.95) | 69.06 (54.42-88.30) | 0.06 (-0.04 to 0.18) | 0.168 (-0.094 to 0.429) |
| **Lao People's Democratic Republic** | 1,536.10 (1,129.00-2,017.61) | 102.38 (75.79-136.34) | 3,842.13 (2,967.60-4,839.35) | 111.23 (87.57-138.44) | 0.09 (-0.1 to 0.32) | 0.271 (0.236 to 0.306) |
| **Lebanon** | 1,614.22 (1,080.04-2,271.15) | 92.94 (60.68-132.36) | 4,546.17 (3,720.37-5,509.35) | 68.90 (56.31-84.18) | -0.26 (-0.49 to 0.12) | -0.992 (-1.105 to -0.879) |
| **Malaysia** | 7,512.88 (5,777.95-9,558.55) | 92.23 (71.37-117.01) | 26,501.53 (20,977.58-32,394.89) | 112.56 (91.20-135.64) | 0.22 (0.1 to 0.39) | 0.614 (0.479 to 0.750) |
| **Maldives** | 57.86 (41.85-76.57) | 97.45 (71.84-126.11) | 261.39 (203.78-337.40) | 93.33 (73.33-118.42) | -0.04 (-0.24 to 0.19) | -0.177 (-0.260 to -0.094) |
| **Mongolia** | 638.56 (483.38-839.15) | 69.37 (53.04-90.70) | 1,287.84 (954.46-1,702.55) | 69.41 (52.42-89.83) | 0 (-0.12 to 0.12) | -0.080 (-0.145 to -0.014) |
| **Myanmar** | 17,968.47 (13,420.05-23,815.51) | 103.21 (77.79-135.89) | 43,946.48 (34,392.53-56,178.18) | 111.54 (88.01-140.63) | 0.08 (-0.14 to 0.31) | 0.252 (0.225 to 0.279) |
| **Nepal** | 4,602.79 (3,202.29-6,368.17) | 66.88 (45.87-93.49) | 16,079.00 (11,992.78-21,120.41) | 87.28 (65.75-116.28) | 0.3 (0.1 to 0.58) | 0.879 (0.828 to 0.930) |
| **Oman** | 412.84 (309.66-525.67) | 80.00 (60.86-102.78) | 1,236.65 (991.36-1,517.64) | 90.96 (74.65-109.22) | 0.14 (-0.17 to 0.54) | 0.238 (-0.041 to 0.517) |
| **Pakistan** | 36,868.24 (26,780.36-49,761.62) | 79.54 (57.90-106.28) | 90,387.22 (69,208.33-116,432.17) | 101.51 (79.65-131.68) | 0.28 (0.1 to 0.51) | 0.771 (0.738 to 0.804) |
| **Palestine** | 582.90 (451.49-730.71) | 87.07 (67.42-108.73) | 1,522.39 (1,248.72-1,837.60) | 89.76 (74.27-107.60) | 0.03 (-0.17 to 0.29) | -0.007 (-0.109 to 0.095) |
| **Philippines** | 23,120.42 (18,497.33-29,118.29) | 111.79 (90.94-136.50) | 72,144.12 (56,994.90-88,258.71) | 110.96 (89.17-135.18) | -0.01 (-0.11 to 0.09) | 0.028 (-0.061 to 0.118) |
| **Qatar** | 62.06 (41.13-78.23) | 116.22 (65.70-150.08) | 407.56 (297.97-555.09) | 78.49 (45.53-103.69) | -0.32 (-0.47 to -0.15) | -1.444 (-2.089 to -0.800) |
| **Republic of Korea** | 22,988.26 (17,735.56-30,412.02) | 93.85 (73.56-124.39) | 89,264.77 (71,028.91-110,071.52) | 96.31 (76.81-117.99) | 0.03 (-0.28 to 0.27) | 0.128 (0.025 to 0.232) |
| **Saudi Arabia** | 2,831.27 (2,244.58-3,620.36) | 64.02 (50.22-81.43) | 9,049.15 (7,157.29-11,409.03) | 72.24 (58.16-88.58) | 0.13 (-0.11 to 0.39) | 0.357 (0.287 to 0.427) |
| **Singapore** | 1,382.68 (1,082.28-1,753.91) | 69.05 (54.34-86.24) | 4,278.48 (3,235.15-5,584.16) | 50.52 (38.39-65.59) | -0.27 (-0.32 to -0.22) | -1.054 (-1.157 to -0.950) |
| **Sri Lanka** | 7,445.73 (5,696.15-9,311.30) | 90.36 (70.80-111.36) | 22,556.30 (16,762.21-28,900.70) | 95.58 (71.97-120.57) | 0.06 (-0.11 to 0.28) | 0.256 (0.069 to 0.442) |
| **Syrian Arab Republic** | 3,246.57 (2,558.49-4,087.47) | 79.86 (62.62-101.42) | 8,108.22 (6,087.08-10,068.96) | 85.65 (63.76-105.23) | 0.07 (-0.15 to 0.39) | 0.066 (-0.171 to 0.304) |
| **Tajikistan** | 1,357.81 (1,008.87-1,881.16) | 55.03 (41.21-75.82) | 2,547.07 (1,826.09-3,444.32) | 54.26 (39.71-72.32) | -0.01 (-0.14 to 0.09) | -0.157 (-0.310 to -0.005) |
| **Thailand** | 30,152.01 (23,731.44-38,013.32) | 112.77 (89.31-141.37) | 110,567.48 (87,501.71-136,070.71) | 101.05 (80.12-124.39) | -0.1 (-0.29 to 0.08) | -0.379 (-0.485 to -0.272) |
| **Timor-Leste** | 178.53 (130.88-232.93) | 90.69 (66.63-119.68) | 691.61 (531.40-906.03) | 102.36 (80.67-131.25) | 0.13 (-0.07 to 0.38) | 0.410 (0.383 to 0.436) |
| **Türkiye** | 19,963.81 (15,940.21-24,778.79) | 72.06 (57.90-89.75) | 55,942.22 (46,417.56-65,879.93) | 67.92 (56.42-80.43) | -0.06 (-0.26 to 0.16) | 0.031 (-0.224 to 0.286) |
| **Turkmenistan** | 1,137.56 (886.98-1,460.32) | 70.46 (55.65-90.88) | 2,974.82 (2,299.06-3,738.29) | 86.50 (67.67-106.69) | 0.23 (0.07 to 0.41) | 0.649 (0.516 to 0.783) |
| **United Arab Emirates** | 231.90 (170.61-301.39) | 79.54 (60.30-104.87) | 1,763.19 (1,272.55-2,341.98) | 85.82 (66.10-105.16) | 0.08 (-0.15 to 0.3) | 0.277 (-0.916 to 1.470) |
| **Uzbekistan** | 5,342.59 (3,748.69-7,548.96) | 50.19 (35.20-71.03) | 12,966.27 (9,656.87-17,427.15) | 58.96 (44.73-77.90) | 0.17 (-0.01 to 0.4) | 0.516 (0.332 to 0.700) |
| **Viet Nam** | 33,050.69 (25,238.73-41,932.36) | 95.92 (73.96-120.29) | 97,148.79 (74,776.52-122,074.21) | 118.87 (91.48-147.64) | 0.24 (0.05 to 0.48) | 0.691 (0.664 to 0.718) |
| **Yemen** | 2,246.10 (1,543.69-3,172.20) | 65.51 (44.69-94.69) | 7,684.86 (5,624.93-9,928.42) | 75.17 (55.62-97.35) | 0.15 (-0.11 to 0.49) | 0.469 (0.398 to 0.539) |

Supplementary Table S6: The decomposition analysis of AF/AFL in Asia, 1990-2021.

| **Location** | **Overll difference** | **Aging (%)** | **Population (%)** | **Epidemiological change (%)** |
| --- | --- | --- | --- | --- |
| **Prevalence** | | | | |
| Central Asia | 194,335.33 | -28,551.24 (-14.69) | 209,235.65 (107.67) | 13,650.92 (7.02) |
| East Asia | 8,220,990.22 | 2,563,272.86 (31.18) | 4,631,302.67 (56.34) | 1,026,414.70 (12.49) |
| High-income Asia Pacific | 1,081,201.62 | 250,866.96 (23.2) | 1,077,691.19 (99.68) | -247,356.52 (-22.88) |
| South Asia | 4,458,601.33 | 1,471,330.65 (33) | 2,892,518.30 (64.88) | 94,752.38 (2.13) |
| Southeast Asia | 2,640,184.64 | 822,117.42 (31.14) | 1,711,854.34 (64.84) | 106,212.88 (4.02) |
| **Incidence** | | | | |
| Central Asia | 17,767.45 | -1,897.91 (-10.68) | 19,222.59 (108.19) | 442.77 (2.49) |
| East Asia | 660,546.11 | 201,799.44 (30.55) | 414,598.90 (62.77) | 44,147.77 (6.68) |
| High-income Asia Pacific | 64,214.21 | 5,981.10 (9.31) | 82,145.68 (127.92) | -23,912.57 (-37.24) |
| South Asia | 443,725.37 | 136,014.48 (30.65) | 299,183.43 (67.43) | 8,527.46 (1.92) |
| Southeast Asia | 243,038.06 | 73,053.01 (30.06) | 164,168.34 (67.55) | 5,816.71 (2.39) |
| **Death** | | | | |
| Central Asia | 836.47 | -85.71 (-10.25) | 672.28 (80.37) | 249.90 (29.88) |
| East Asia | 61,565.84 | 38,480.75 (62.5) | 30,112.03 (48.91) | -7,026.94 (-11.41) |
| High-income Asia Pacific | 10,968.64 | 6,515.46 (59.4) | 6,744.18 (61.49) | -2,291.01 (-20.89) |
| South Asia | 28,135.65 | 8,683.26 (30.86) | 12,326.98 (43.81) | 7,125.41 (25.33) |
| Southeast Asia | 19,537.39 | 6,901.18 (35.32) | 9,625.87 (49.27) | 3,010.35 (15.41) |
| **DALYs** | | | | |
| Central Asia | 28,094.05 | -3,639.23 (-12.95) | 27,055.82 (96.3) | 4,677.46 (16.65) |
| East Asia | 1,315,757.96 | 592,980.02 (45.07) | 753,196.73 (57.24) | -30,418.79 (-2.31) |
| High-income Asia Pacific | 196,771.83 | 76,956.76 (39.11) | 170,365.81 (86.58) | -50,550.73 (-25.69) |
| South Asia | 749,013.91 | 225,040.96 (30.04) | 417,102.55 (55.69) | 106,870.40 (14.27) |
| Southeast Asia | 450,297.21 | 142,746.57 (31.7) | 265,581.13 (58.98) | 41,969.50 (9.32) |

Supplementary Table S7: The decomposition analysis of AF/AFL in Asia, 1990-2013.

| **Location** | **Overll difference** | **Aging (%)** | **Population (%)** | **Epidemiological change (%)** |
| --- | --- | --- | --- | --- |
| **Prevalence** | | | | |
| Central Asia | 105,510.04 | -45,629.27 (-43.25) | 142,940.14 (135.48) | 8,199.17 (7.77) |
| East Asia | 449,2397.5 | 1,319,187.75 (29.36) | 4,190,744.67 (93.29) | -1,017,534.92 (-22.65) |
| High-income Asia Pacific | 559,786.13 | 206,986.01 (36.98) | 725,215.54 (129.55) | -372415.42 (-66.53) |
| South Asia | 2,896,652.92 | 867,996.62 (29.97) | 1,941,824.32 (67.04) | 86,831.98 (3) |
| Southeast Asia | 1,623,547.05 | 418,919.95 (25.8) | 1,130,959.09 (69.66) | 73,668.01 (4.54) |
| **Incidence** | | | | |
| Central Asia | 8,778.86 | -4,356.4 (-49.62) | 12,956.59 (147.59) | 178.66 (2.04) |
| East Asia | 364,854.99 | 103,366.32 (28.33) | 263,672.14 (72.27) | -2,183.47 (-0.6) |
| High-income Asia Pacific | 29,874 | 7,390.85 (24.74) | 55,950.8 (187.29) | -33,467.66 (-112.03) |
| South Asia | 289,326 | 79,186.45 (27.37) | 201,970.84 (69.81) | 8,168.72 (2.82) |
| Southeast Asia | 151,436.61 | 37,776.82 (24.95) | 109,385.32 (72.23) | 4,274.48 (2.82) |
| **Death** | | | | |
| Central Asia | 471.93 | -140.56 (-29.79) | 446.58 (94.63) | 165.91 (35.16) |
| East Asia | 32,340.34 | 19,490.1 (60.27) | 17,587.63 (54.38) | -4,737.39 (-14.65) |
| High-income Asia Pacific | 6,566.01 | 4,430.16 (67.47) | 4,367.31 (66.51) | -2,231.46 (-33.99) |
| South Asia | 17,374.61 | 5,105.5 (29.38) | 7,737.8 (44.54) | 4,531.3 (26.08) |
| Southeast Asia | 12,108.19 | 3,856.24 (31.85) | 6,146.42 (50.76) | 2,105.52 (17.39) |
| **DALYs** | | | | |
| Central Asia | 15,774.17 | -5,876.53 (-37.25) | 18,369.01 (116.45) | 3,281.69 (20.8) |
| East Asia | 720,750.36 | 298,937.55 (41.48) | 467,816.81 (64.91) | -46,004 (-6.38) |
| High-income Asia Pacific | 113,656.52 | 57,118.67 (50.26) | 11,4835.78 (101.04) | -58,297.93 (-51.29) |
| South Asia | 468,254.6 | 130,399.18 (27.85) | 272,313.04 (58.15) | 65,542.38 (14) |
| Southeast Asia | 279,868.26 | 75,926.41 (27.13) | 174,094.69 (62.21) | 29,847.17 (10.66) |

Supplementary Table S8: The decomposition analysis of AF/AFL in Asia, 2014-2021.

| **Location** | **Overll difference** | **Aging (%)** | **Population (%)** | **Epidemiological change (%)** |
| --- | --- | --- | --- | --- |
| **Prevalence** | | | | |
| Central Asia | 81,294.51 | 26,935.51 (33.13) | 49,293.39 (60.64) | 5,065.61 (6.23) |
| East Asia | 3,376,580.82 | 1,206,017.66 (35.72) | 1,334,842.15 (39.53) | 835,721.02 (24.75) |
| High-income Asia Pacific | 474,891.85 | -9,545.18 (-2.01) | 255,238.93 (53.75) | 229,198.09 (48.26) |
| South Asia | 1,387,331.61 | 568,878.14 (41.01) | 824,297.97 (59.42) | -5,844.5 (-0.42) |
| Southeast Asia | 910,365.52 | 405,669.51 (44.56) | 481,709.99 (52.91) | 22,986.01 (2.52) |
| **Incidence** | | | | |
| Central Asia | 8,183.48 | 3,398.14 (41.52) | 4,471.24 (54.64) | 314.1 (3.84) |
| East Asia | 267,840.83 | 84,586.95 (31.58) | 114,670.06 (42.81) | 68,583.81 (25.61) |
| High-income Asia Pacific | 31,208.42 | -3,836.71 (-12.29) | 18,194.78 (58.3) | 16,850.35 (53.99) |
| South Asia | 136,806.71 | 53,610.48 (39.19) | 84,145.94 (61.51) | -949.71 (-0.69) |
| Southeast Asia | 81,925.23 | 35,140.66 (42.89) | 45,623.73 (55.69) | 1,160.85 (1.42) |
| **Death** | | | | |
| Central Asia | 203.93 | 98.35 (48.23) | 181.36 (88.93) | -75.78 (-37.16) |
| East Asia | 2,6331.11 | 17,551.02 (66.66) | 8,983.25 (34.12) | -203.16 (-0.77) |
| High-income Asia Pacific | 3,972.2 | 1,451.8 (36.55) | 1,889.56 (47.57) | 630.83 (15.88) |
| South Asia | 8,124.31 | 3,705.36 (45.61) | 4,253.69 (52.36) | 165.26 (2.03) |
| Southeast Asia | 6,754.65 | 3,046.6 (45.1) | 3,041.63 (45.03) | 666.43 (9.87) |
| **DALYs** | | | | |
| Central Asia | 9,086.76 | 3,702.34 (40.74) | 6,743.09 (74.21) | -1,358.68 (-14.95) |
| East Asia | 537,823.11 | 269,461.13 (50.1) | 214,608.25 (39.9) | 53,753.73 (9.99) |
| High-income Asia Pacific | 75,807.53 | 9,705.58 (12.8) | 42,523.1 (56.09) | 23,578.84 (31.1) |
| South Asia | 237,232.65 | 92,115.91 (38.83) | 126,619.2 (53.37) | 18,497.54 (7.8) |
| Southeast Asia | 153,781.37 | 67,089.59 (43.63) | 77,763.45 (50.57) | 8,928.33 (5.81) |

Supplementary Table S9: Risk factors of AF/AFL in Asia, 1990 to 2021.

|  | **Diet high in sodium** | **High alcohol use** | **High body-mass index** | **High systolic blood pressure** | **Other environmental risks** | **Smoking** |
| --- | --- | --- | --- | --- | --- | --- |
| **ASMR 100,000 (95% UI)** | | | | | | |
| 1990 | 0.1527 (0.0194-0.4214) | 0.0687 (0.0487-0.0923) | 0.0353 (0.0131-0.0620) | 1.0494 (0.3786-1.7518) | 0.1014 (0-0.2629) | 0.1615 (0.0893-0.2478) |
| 1991 | 0.1541 (0.0188-0.4237) | 0.0702 (0.0508-0.0920) | 0.0368 (0.0139-0.0642) | 1.0590 (0.3807-1.7640) | 0.1033 (0-0.2711) | 0.1627 (0.0924-0.2434) |
| 1992 | 0.1556 (0.0192-0.4230) | 0.0716 (0.0517-0.0939) | 0.0385 (0.0146-0.0676) | 1.0754 (0.3914-1.7900) | 0.1058 (0-0.2782) | 0.1641 (0.0915-0.2508) |
| 1993 | 0.1560 (0.0189-0.4242) | 0.0730 (0.0531-0.0952) | 0.0401 (0.0151-0.0704) | 1.0879 (0.3885-1.8083) | 0.1076 (0-0.2766) | 0.1652 (0.0915-0.2516) |
| 1994 | 0.1559 (0.0185-0.4302) | 0.0742 (0.0546-0.0967) | 0.0417 (0.0156-0.0715) | 1.1018 (0.4011-1.8088) | 0.1098 (0-0.2842) | 0.1661 (0.0932-0.2520) |
| 1995 | 0.1571 (0.0180-0.4335) | 0.0757 (0.0557-0.0989) | 0.0436 (0.0164-0.0746) | 1.1255 (0.4072-1.8446) | 0.1131 (0-0.2915) | 0.1676 (0.0949-0.2528) |
| 1996 | 0.1565 (0.0182-0.4360) | 0.0760 (0.0565-0.0996) | 0.0451 (0.0171-0.0779) | 1.1300 (0.4019-1.8506) | 0.1142 (0-0.2984) | 0.1671 (0.0950-0.2530) |
| 1997 | 0.1554 (0.0170-0.4309) | 0.0754 (0.0555-0.0979) | 0.0463 (0.0176-0.0795) | 1.1285 (0.4004-1.8479) | 0.1146 (0-0.2912) | 0.1657 (0.0940-0.2490) |
| 1998 | 0.1553 (0.0172-0.4367) | 0.0751 (0.0557-0.0970) | 0.0480 (0.0185-0.0827) | 1.1354 (0.4061-1.8997) | 0.1159 (0-0.2958) | 0.1645 (0.0942-0.2455) |
| 1999 | 0.1550 (0.0167-0.4345) | 0.0745 (0.0554-0.0962) | 0.0500 (0.0193-0.0869) | 1.1383 (0.4027-1.8774) | 0.1167 (0-0.2977) | 0.1622 (0.0944-0.2410) |
| 2000 | 0.1546 (0.0165-0.4372) | 0.0741 (0.0550-0.0957) | 0.0523 (0.0205-0.0900) | 1.1418 (0.4075-1.8676) | 0.1179 (0-0.2920) | 0.1611 (0.0942-0.2379) |
| 2001 | 0.1530 (0.0158-0.4383) | 0.0733 (0.0551-0.0943) | 0.0546 (0.0215-0.0930) | 1.1451 (0.4064-1.8714) | 0.1197 (0-0.3047) | 0.1591 (0.0925-0.2363) |
| 2002 | 0.1487 (0.0147-0.4238) | 0.0712 (0.0531-0.0921) | 0.0568 (0.0224-0.0971) | 1.1293 (0.3948-1.8486) | 0.1204 (0-0.3016) | 0.1550 (0.0914-0.2275) |
| 2003 | 0.1476 (0.0147-0.4256) | 0.0700 (0.0518-0.0899) | 0.0592 (0.0232-0.1005) | 1.1313 (0.3881-1.8499) | 0.1235 (0-0.3050) | 0.1538 (0.0893-0.2265) |
| 2004 | 0.1471 (0.0143-0.4254) | 0.0691 (0.0517-0.0884) | 0.0619 (0.0244-0.1050) | 1.1371 (0.3863-1.8573) | 0.1261 (0-0.3185) | 0.1519 (0.0876-0.2258) |
| 2005 | 0.1446 (0.0140-0.4202) | 0.0681 (0.0507-0.0873) | 0.0644 (0.0257-0.1101) | 1.1255 (0.3821-1.8359) | 0.1253 (0-0.3158) | 0.1482 (0.0860-0.2188) |
| 2006 | 0.1398 (0.0132-0.4076) | 0.0674 (0.0501-0.0866) | 0.0665 (0.0266-0.1121) | 1.1000 (0.3691-1.8077) | 0.1236 (0-0.3141) | 0.1441 (0.0839-0.2122) |
| 2007 | 0.1391 (0.0132-0.4069) | 0.0691 (0.0515-0.0887) | 0.0697 (0.0280-0.1199) | 1.1008 (0.3723-1.8063) | 0.1249 (0-0.3118) | 0.1436 (0.0823-0.2114) |
| 2008 | 0.1400 (0.0130-0.4131) | 0.0715 (0.0535-0.0917) | 0.0734 (0.0293-0.1251) | 1.1108 (0.3758-1.8239) | 0.1263 (0-0.3161) | 0.1430 (0.0821-0.2115) |
| 2009 | 0.1407 (0.0130-0.4077) | 0.0736 (0.0558-0.0944) | 0.0769 (0.0306-0.1300) | 1.1140 (0.3722-1.8160) | 0.1267 (0-0.3250) | 0.1427 (0.0826-0.2121) |
| 2010 | 0.1414 (0.0130-0.4139) | 0.0757 (0.0561-0.0972) | 0.0806 (0.0322-0.1373) | 1.1219 (0.3810-1.8399) | 0.1277 (0-0.3199) | 0.1430 (0.0814-0.2107) |
| 2011 | 0.1397 (0.0134-0.4060) | 0.0764 (0.0573-0.0969) | 0.0843 (0.0339-0.1418) | 1.1160 (0.3768-1.8248) | 0.1270 (0-0.3164) | 0.1407 (0.0811-0.2059) |
| 2012 | 0.1368 (0.0129-0.4051) | 0.0768 (0.0577-0.0975) | 0.0870 (0.0350-0.1453) | 1.0995 (0.3754-1.8238) | 0.1258 (0-0.3118) | 0.1379 (0.0808-0.2016) |
| 2013 | 0.1368 (0.0131-0.4034) | 0.0779 (0.0590-0.0997) | 0.0915 (0.0370-0.1542) | 1.1167 (0.3842-1.8534) | 0.1297 (0-0.3231) | 0.1382 (0.0799-0.2031) |
| 2014 | 0.1378 (0.0129-0.3986) | 0.0792 (0.0598-0.1008) | 0.0972 (0.0393-0.1623) | 1.1358 (0.3953-1.8784) | 0.1333 (0-0.3298) | 0.1388 (0.0790-0.2040) |
| 2015 | 0.1370 (0.0128-0.4143) | 0.0797 (0.0601-0.1028) | 0.1014 (0.0406-0.1690) | 1.1284 (0.3950-1.8846) | 0.1318 (0-0.3309) | 0.1368 (0.0779-0.1994) |
| 2016 | 0.1376 (0.0123-0.4083) | 0.0801 (0.0602-0.1032) | 0.1066 (0.0432-0.1757) | 1.1304 (0.3891-1.8936) | 0.1313 (0-0.3288) | 0.1354 (0.0783-0.1990) |
| 2017 | 0.1386 (0.0131-0.4249) | 0.0806 (0.0609-0.1044) | 0.1125 (0.0451-0.1884) | 1.1401 (0.3934-1.9039) | 0.1319 (0-0.3320) | 0.1346 (0.0794-0.1981) |
| 2018 | 0.1388 (0.0127-0.4161) | 0.0808 (0.0613-0.1043) | 0.1177 (0.0482-0.1995) | 1.1398 (0.3928-1.8819) | 0.1310 (0-0.3328) | 0.1334 (0.0774-0.1955) |
| 2019 | 0.1398 (0.0130-0.4317) | 0.0817 (0.0611-0.1064) | 0.1244 (0.0509-0.2113) | 1.1505 (0.3948-1.8965) | 0.1319 (0-0.3329) | 0.1336 (0.0780-0.1981) |
| 2020 | 0.1392 (0.0128-0.4194) | 0.0812 (0.0594-0.1060) | 0.1290 (0.0526-0.2194) | 1.1424 (0.3921-1.9017) | 0.1311 (0-0.3293) | 0.1325 (0.0759-0.1973) |
| 2021 | 0.1400 (0.0136-0.4160) | 0.0824 (0.0613-0.1096) | 0.1364 (0.0559-0.2267) | 1.1455 (0.3951-1.8903) | 0.1304 (0-0.3214) | 0.1322 (0.0767-0.1922) |
| **Age‑standardized DALYs rate 100,000 (95% UI)** | | | | | | |
| 1990 | 4.1592 (0.6547-10.5711) | 2.1022 (1.4671-2.8167) | 0.8674 (0.3214-1.4831) | 24.0624 (8.2980-40.0020) | 2.6192 (0-6.7270) | 5.5308 (3.2663-8.1626) |
| 1991 | 4.1585 (0.6539-10.5710) | 2.1114 (1.5014-2.8008) | 0.9034 (0.3367-1.5286) | 24.1399 (8.3384-40.2243) | 2.6484 (0-6.6331) | 5.4973 (3.2089-8.1957) |
| 1992 | 4.1589 (0.6501-10.5443) | 2.1183 (1.4892-2.8002) | 0.9420 (0.3510-1.5936) | 24.2958 (8.3576-40.5442) | 2.6820 (0-6.7940) | 5.4654 (3.1686-8.0858) |
| 1993 | 4.1348 (0.6376-10.6421) | 2.1241 (1.4845-2.8140) | 0.9811 (0.3674-1.6593) | 24.4187 (8.3225-40.7815) | 2.7084 (0-6.9738) | 5.4319 (3.1680-8.0393) |
| 1994 | 4.1070 (0.6276-10.4699) | 2.1289 (1.5017-2.8364) | 1.0220 (0.3853-1.7198) | 24.5748 (8.4119-40.7150) | 2.7401 (0-6.9075) | 5.3982 (3.1622-8.0558) |
| 1995 | 4.1007 (0.6193-10.4483) | 2.1438 (1.5121-2.8338) | 1.0668 (0.4031-1.8001) | 24.8581 (8.5877-41.4110) | 2.7845 (0-6.9816) | 5.3813 (3.2007-8.0141) |
| 1996 | 4.0698 (0.5967-10.4779) | 2.1341 (1.5071-2.8166) | 1.1087 (0.4192-1.8606) | 24.9053 (8.4301-41.2927) | 2.8031 (0-7.0653) | 5.3319 (3.1734-7.9265) |
| 1997 | 4.0288 (0.5935-10.5009) | 2.1051 (1.4975-2.7621) | 1.1489 (0.4326-1.9368) | 24.8772 (8.4349-40.9267) | 2.8109 (0-7.1027) | 5.2630 (3.1028-7.8403) |
| 1998 | 4.0018 (0.5705-10.4467) | 2.0779 (1.4849-2.7398) | 1.1984 (0.4498-2.0192) | 24.9418 (8.4996-41.5028) | 2.8311 (0-7.1507) | 5.1989 (3.0852-7.7960) |
| 1999 | 3.9775 (0.5708-10.4665) | 2.0499 (1.4667-2.6927) | 1.2523 (0.4663-2.1249) | 24.9597 (8.4128-41.2639) | 2.8448 (0-7.1779) | 5.1273 (3.0329-7.6451) |
| 2000 | 3.9545 (0.5622-10.4943) | 2.0356 (1.4441-2.6828) | 1.3098 (0.4871-2.2170) | 24.9919 (8.4429-41.2486) | 2.8625 (0-7.2975) | 5.0782 (3.0041-7.6132) |
| 2001 | 3.9339 (0.5445-10.5233) | 2.0309 (1.4516-2.6630) | 1.3736 (0.5121-2.3231) | 25.0889 (8.5415-41.5295) | 2.8936 (0-7.3553) | 5.0413 (2.9802-7.5322) |
| 2002 | 3.9051 (0.5298-10.4723) | 2.0246 (1.4438-2.6703) | 1.4442 (0.5409-2.4217) | 25.0637 (8.3811-41.1897) | 2.9233 (0-7.4329) | 5.0058 (2.9680-7.4796) |
| 2003 | 3.9199 (0.5257-10.5158) | 2.0325 (1.4455-2.6925) | 1.5240 (0.5736-2.5546) | 25.2451 (8.3699-41.6714) | 2.9774 (0-7.5969) | 5.0103 (2.9497-7.5076) |
| 2004 | 3.9407 (0.5257-10.5410) | 2.0453 (1.4530-2.7137) | 1.6080 (0.6095-2.6871) | 25.4621 (8.5183-41.9624) | 3.0229 (0-7.6186) | 5.0088 (2.9295-7.4875) |
| 2005 | 3.9178 (0.5273-10.4390) | 2.0468 (1.4555-2.7121) | 1.6872 (0.6433-2.8036) | 25.4270 (8.4436-41.8302) | 3.0207 (0-7.6494) | 4.9613 (2.9181-7.4729) |
| 2006 | 3.8503 (0.5160-10.3454) | 2.0524 (1.4676-2.7367) | 1.7593 (0.6757-2.9123) | 25.1661 (8.3024-41.2432) | 2.9988 (0-7.6563) | 4.8936 (2.8565-7.3037) |
| 2007 | 3.8267 (0.5190-10.3837) | 2.1012 (1.4933-2.7993) | 1.8502 (0.7147-3.0385) | 25.1637 (8.2490-41.2245) | 3.0098 (0-7.6613) | 4.8762 (2.8394-7.3042) |
| 2008 | 3.8237 (0.5127-10.3466) | 2.1657 (1.5376-2.8839) | 1.9493 (0.7611-3.2023) | 25.2627 (8.2986-41.1695) | 3.0196 (0-7.6477) | 4.8610 (2.8221-7.1995) |
| 2009 | 3.8239 (0.5062-10.2917) | 2.2278 (1.6050-2.9590) | 2.0476 (0.8078-3.3560) | 25.2961 (8.2226-41.3674) | 3.0208 (0-7.5930) | 4.8506 (2.8544-7.1591) |
| 2010 | 3.8265 (0.5111-10.1859) | 2.2802 (1.6345-3.0207) | 2.1507 (0.8452-3.5031) | 25.4204 (8.3642-41.6174) | 3.0298 (0-7.7208) | 4.8437 (2.8029-7.1938) |
| 2011 | 3.8006 (0.5068-10.2781) | 2.3082 (1.6616-3.0967) | 2.2547 (0.8823-3.6938) | 25.3720 (8.2384-41.6400) | 3.0203 (0-7.5898) | 4.8024 (2.8195-7.1346) |
| 2012 | 3.7611 (0.4964-10.2099) | 2.3286 (1.6839-3.0861) | 2.3516 (0.9208-3.8528) | 25.1963 (8.2610-41.6474) | 2.9993 (0-7.6664) | 4.7481 (2.7673-7.0098) |
| 2013 | 3.7467 (0.4973-10.2195) | 2.3528 (1.6883-3.1173) | 2.4697 (0.9627-4.0215) | 25.3602 (8.3227-41.9735) | 3.0263 (0-7.6984) | 4.7186 (2.7502-7.0347) |
| 2014 | 3.7464 (0.4980-10.0036) | 2.3781 (1.7174-3.1658) | 2.6034 (1.0349-4.2352) | 25.5365 (8.3612-42.4088) | 3.0477 (0-7.8096) | 4.6958 (2.7429-6.9251) |
| 2015 | 3.7365 (0.5103-10.0604) | 2.3963 (1.7277-3.1740) | 2.7249 (1.0729-4.4651) | 25.5016 (8.3589-42.2096) | 3.0263 (0-7.7889) | 4.6597 (2.7319-6.9013) |
| 2016 | 3.7524 (0.5086-10.2404) | 2.4165 (1.7342-3.1775) | 2.8620 (1.1436-4.6865) | 25.5992 (8.4475-42.7887) | 3.0181 (0-7.8808) | 4.6389 (2.6909-6.8556) |
| 2017 | 3.7844 (0.5062-10.5601) | 2.4448 (1.7618-3.2189) | 3.0186 (1.2018-4.9729) | 25.8573 (8.4274-43.0935) | 3.0286 (0-7.7905) | 4.6347 (2.7118-6.8309) |
| 2018 | 3.8070 (0.5117-10.4792) | 2.4698 (1.7881-3.2653) | 3.1695 (1.2509-5.2789) | 26.0035 (8.5902-43.4037) | 3.0210 (0-7.8409) | 4.6243 (2.6890-6.8879) |
| 2019 | 3.8301 (0.5064-10.6513) | 2.4963 (1.7911-3.3256) | 3.3316 (1.3343-5.5908) | 26.1979 (8.5121-43.7036) | 3.0218 (0-7.7379) | 4.6226 (2.7012-6.8838) |
| 2020 | 3.8229 (0.5261-10.2343) | 2.4945 (1.7852-3.2896) | 3.4599 (1.3843-5.6580) | 26.0947 (8.4870-43.2506) | 2.9935 (0-7.7552) | 4.5855 (2.6655-6.8259) |
| 2021 | 3.8675 (0.5472-10.5522) | 2.5298 (1.8127-3.3391) | 3.6559 (1.4459-6.1062) | 26.2632 (8.6839-43.8824) | 2.9812 (0-7.5770) | 4.5993 (2.6726-6.8054) |

Supplementary Table S10. Global disease burden prediction for AF/AFL in Asia to 2040.

|  | **Both** | | | | **Female** | | | | **Male** | | | |
| --- | --- | --- | --- | --- | --- | --- | --- | --- | --- | --- | --- | --- |
|  | **Prevalence (95% UI)** | **Incidence (95% UI)** | **Death (95% UI)** | **DALYs (95% UI)** | **Prevalence (95% UI)** | **Incidence (95% UI)** | **Death (95% UI)** | **DALYs (95% UI)** | **Prevalence (95% UI)** | **Incidence (95% UI)** | **Death (95% UI)** | **DALYs (95% UI)** |
| **2022** | 26,876,565.71 (26,591,498.18-27,164,689.23) | 2,409,267.08 (2,387,828.77-2,430,897.87) | 160,779.57 (158,106.04-163,498.31) | 4,198,987.71 (4,150,850.53-4,247,683.14) | 13,140,177.08 (13,005,631.16-13,276,114.90) | 1,212,574.61 (1,201,060.11-1,224,199.50) | 96,940.48 (95,208.90-98,703.55) | 2,197,699.11 (2,170,191.82-2,225,555.05) | 13,919,459.96 (13,772,690.93-14,067,793.05) | 1,200,162.43 (1,190,047.04-1,210,363.80) | 63,875.34 (62,748.10-65,022.82) | 2,004,287.22 (1,982,528.61-2,026,284.63) |
| **2023** | 28,064,306.62 (27,403,066.15-28,741,502.94) | 2,505,468.35 (2,455,890.66-2,556,046.89) | 168,006.34 (162,810.82-173,367.65) | 4,368,301.00 (4,272,303.27-4,466,455.77) | 13,995,473.42 (13,557,275.90-14,447,834.35) | 1,271,275.00 (1,244,439.68-1,298,689.00) | 101,113.90 (98,028.48-104,296.44) | 2,291,517.98 (2,232,847.03-2,351,730.60) | 14,456,961.31 (14,118,322.26-14,803,722.88) | 1,241,528.16 (1,214,264.36-1,269,404.13) | 66,757.67 (64,650.46-68,933.56) | 2,080,883.67 (2,039,866.09-2,122,726.02) |
| **2024** | 29,304,536.71 (28,158,360.64-30,497,367.47) | 2,605,510.91 (2,519,814.99-2,694,121.25) | 175,557.93 (168,482.34-182,930.67) | 4,544,441.40 (4,405,916.83-4,687,321.27) | 14,906,441.15 (14,049,831.89-15,815,277.34) | 1,332,817.06 (1,286,074.52-1,381,258.47) | 105,641.79 (101,796.92-109,631.88) | 2,389,341.95 (2,303,293.98-2,478,604.55) | 15,015,218.33 (14,431,338.55-15,622,721.38) | 1,284,319.65 (1,234,655.44-1,335,981.60) | 69,770.07 (66,919.22-72,742.36) | 2,160,407.35 (2,101,986.80-2,220,451.58) |
| **2025** | 30,599,575.58 (28,863,603.53-32,439,955.90) | 2,709,548.14 (2,580,094.18-2,845,497.34) | 183,448.96 (174,682.11-192,655.80) | 4,727,684.21 (4,545,772.64-4,916,875.47) | 15,876,703.92 (14,489,219.87-17,397,053.09) | 1,397,338.36 (1,326,189.09-1,472,304.74) | 110,372.43 (105,801.86-115,140.44) | 2,491,341.98 (2,377,249.77-2,610,909.85) | 15,595,032.49 (14,715,377.67-16,527,271.25) | 1,328,586.01 (1,251,744.07-1,410,145.13) | 72,918.39 (69,395.45-76,620.18) | 2,242,970.13 (2,166,780.18-2,321,839.12) |
| **2026** | 31,951,845.38 (29,522,407.69-34,581,204.68) | 2,817,739.54 (2,637,016.78-3,010,847.78) | 191,694.67 (181,307.53-202,676.90) | 4,918,315.81 (4,690,428.73-5,157,274.91) | 16,910,121.26 (14,878,840.65-19,218,715.20) | 1,464,983.11 (1,364,903.39-1,572,401.04) | 115,314.91 (110,027.79-120,856.09) | 2,597,696.34 (2,453,733.24-2,750,105.92) | 16,197,236.24 (14,972,689.54-17,521,932.92) | 1,374,378.10 (1,265,847.29-1,492,214.09) | 76,208.79 (72,040.48-80,618.28) | 2,328,688.15 (2,233,700.34-2,427,715.30) |
| **2027** | 33,363,875.27 (30,136,990.18-36,936,275.52) | 2,930,250.99 (2,690,761.02-3,191,056.66) | 200,311.02 (188,322.01-213,063.27) | 5,116,634.13 (4,839,327.01-5,409,831.74) | 18,010,803.91 (15,220,714.42-21,312,341.08) | 1,535,902.53 (1,402,280.88-1,682,256.81) | 120,478.71 (114,470.51-126,802.26) | 2,708,590.94 (2,532,363.35-2,897,082.23) | 16,822,694.15 (15,204,780.37-18,612,767.26) | 1,421,748.49 (1,277,185.82-1,582,673.99) | 79,647.66 (74,840.42-84,763.69) | 2,417,682.00 (2,302,534.67-2,538,587.73) |
| **2028** | 34,838,306.20 (30,708,788.84-39,523,134.08) | 3,047,254.99 (2,741,442.32-3,387,181.58) | 209,314.65 (195,712.59-223,862.06) | 5,322,949.13 (4,992,214.64-5,675,594.79) | 19,183,130.17 (15,516,205.25-23,716,654.76) | 1,610,255.13 (1,438,352.88-1,802,701.99) | 125,873.75 (119,131.08-132,998.05) | 2,824,219.57 (2,612,960.15-3,052,559.45) | 17,472,304.18 (15,412,739.37-19,807,083.34) | 1,470,751.59 (1,285,929.05-1,682,138.10) | 83,241.71 (77,790.44-89,074.99) | 2,510,076.87 (2,373,188.84-2,654,860.74) |
| **2029** | 36,377,895.83 (31,238,784.87-42,362,444.97) | 3,168,930.91 (2,789,138.32-3,600,439.27) | 218,722.98 (203,477.33-235,110.93) | 5,537,583.24 (5,148,973.21-5,955,522.96) | 20,431,763.35 (15,766,377.52-26,477,670.80) | 1,688,207.12 (1,473,131.31-1,934,683.80) | 131,510.38 (124,013.45-139,460.53) | 2,944,784.35 (2,695,430.41-3,217,205.99) | 18,146,998.96 (15,597,409.52-21,113,350.32) | 1,521,443.67 (1,292,216.70-1,791,333.33) | 86,997.94 (80,889.96-93,567.14) | 2,606,002.72 (2,445,621.49-2,776,901.58) |
| **2030** | 37,985,523.67 (31,727,685.53-45,477,632.06) | 3,295,465.32 (2,833,903.00-3,832,203.04) | 228,554.20 (211,620.34-246,843.12) | 5,760,871.92 (5,309,552.59-6,250,554.03) | 21,761,670.28 (15,972,183.65-29,649,689.97) | 1,769,932.75 (1,506,616.06-2,079,270.24) | 137,399.42 (129,123.41-146,205.88) | 3,070,495.99 (2,779,724.15-3,391,683.89) | 18,847,747.15 (15,759,484.04-22,541,193.09) | 1,573,882.94 (1,296,170.44-1,911,097.05) | 90,923.67 (84,140.75-98,253.38) | 2,705,594.51 (2,519,818.51-2,905,067.01) |
| **2031** | 39,664,196.51 (32,176,034.70-48,895,039.41) | 3,427,052.21 (2,875,775.36-4,084,007.06) | 238,827.32 (220,149.56-259,089.73) | 5,993,164.13 (5,473,940.82-6,561,637.69) | 23,178,141.07 (16,134,568.55-33,296,596.79) | 1,855,614.69 (1,538,799.36-2,237,657.47) | 143,552.17 (134,468.06-153,249.97) | 3,201,574.20 (2,865,815.08-3,576,670.88) | 19,575,554.80 (15,899,564.46-24,101,436.67) | 1,628,129.62 (1,297,900.61-2,042,379.86) | 95,026.54 (87,546.07-103,146.18) | 2,808,992.33 (2,595,781.20-3,039,716.10) |
| **2032** | 41,417,053.99 (32,584,283.16-52,644,164.44) | 3,563,893.32 (2,914,784.91-4,357,555.02) | 249,562.20 (229,075.52-271,881.04) | 6,234,822.93 (5,642,148.83-6,889,753.91) | 24,686,810.18 (16,254,529.36-37,493,463.12) | 1,945,444.47 (1,569,668.68-2,411,180.29) | 149,980.44 (140,055.49-160,608.72) | 3,338,248.09 (2,953,690.79-3,772,873.03) | 20,331,466.82 (16,018,197.28-25,806,183.80) | 1,684,246.02 (1,297,510.33-2,186,252.06) | 99,314.55 (91,110.22-108,257.66) | 2,916,341.63 (2,673,520.19-3,181,217.23) |
| **2033** | 43,247,374.51 (32,952,835.06-56,757,951.13) | 3,706,198.46 (2,950,955.43-4,654,732.12) | 260,779.59 (238,410.74-285,247.20) | 6,486,225.99 (5,814,202.00-7,235,924.64) | 26,293,678.82 (16,333,149.17-42,328,490.30) | 2,039,622.88 (1,599,208.68-2,601,324.99) | 156,696.57 (145,894.63-168,298.28) | 3,480,756.53 (3,043,347.32-3,981,032.98) | 21,116,568.46 (16,115,897.90-27,668,918.38) | 1,742,296.56 (1,295,098.05-2,343,913.11) | 103,796.06 (94,838.26-113,599.94) | 3,027,793.42 (2,753,052.12-3,329,952.58) |
| **2034** | 45,158,581.35 (33,282,079.48-61,273,138.62) | 3,854,185.79 (2,984,307.45-4,977,619.89) | 272,501.18 (248,169.30-299,218.70) | 6,747,766.22 (5,990,135.20-7,601,222.26) | 28,005,138.82 (16,371,614.93-47,905,341.25) | 2,138,360.45 (1,627,402.53-2,809,744.55) | 163,713.44 (151,995.13-176,335.20) | 3,629,348.60 (3,134,786.08-4,201,936.26) | 21,931,986.88 (16,193,166.60-29,704,631.61) | 1,802,347.92 (1,290,759.20-2,516,703.36) | 108,479.79 (98,735.89-119,185.27) | 3,143,504.49 (2,834,397.62-3,486,321.18) |
| **2035** | 47,154,249.08 (33,572,412.25-66,230,665.52) | 4,008,082.21 (3,014,860.10-5,328,513.59) | 284,749.64 (258,366.67-313,826.69) | 7,019,852.39 (6,169,989.73-7,986,776.27) | 29,827,998.03 (16,371,225.02-54,345,931.06) | 2,241,877.87 (1,654,232.94-3,038,276.07) | 171,044.54 (158,367.33-184,736.55) | 3,784,284.01 (3,228,011.90-4,436,416.56) | 22,778,892.76 (16,250,499.39-31,929,969.82) | 1,864,469.05 (1,284,587.24-2,706,118.16) | 113,374.87 (102,809.35-125,026.17) | 3,263,637.61 (2,917,580.14-3,650,741.34) |
| **2036** | 49,238,110.23 (33,824,251.18-71,676,132.19) | 4,168,123.67 (3,042,632.39-5,709,942.13) | 297,548.64 (269,019.55-329,103.20) | 7,302,909.72 (6,353,811.44-8,393,779.22) | 31,769,507.46 (16,333,389.98-61,793,761.42) | 2,350,406.54 (1,679,682.83-3,288,960.75) | 178,703.91 (165,022.19-193,519.97) | 3,945,833.54 (3,323,031.86-4,685,360.54) | 23,658,501.99 (16,288,395.35-34,363,404.40) | 1,928,731.31 (1,276,674.33-2,913,824.11) | 118,490.83 (107,065.38-131,135.55) | 3,388,361.77 (3,002,625.09-3,823,652.67) |
| **2037** | 51,414,062.28 (34,038,046.69-77,660,325.94) | 4,334,555.53 (3,067,644.15-6,124,690.70) | 310,922.94 (280,145.76-345,081.34) | 7,597,380.61 (6,541,649.36-8,823,492.20) | 33,837,390.06 (16,259,628.55-70,417,904.24) | 2,464,189.05 (1,703,735.88-3,564,066.32) | 186,706.28 (171,971.31-202,703.78) | 4,114,279.56 (3,419,854.42-4,949,712.53) | 24,572,077.43 (16,307,361.50-37,025,424.95) | 1,995,208.47 (1,267,111.74-3,141,677.82) | 123,837.66 (111,511.16-137,526.73) | 3,517,852.42 (3,089,559.41-4,005,517.95) |
| **2038** | 53,686,174.96 (34,214,289.06-84,239,815.02) | 4,507,632.96 (3,089,916.71-6,575,826.08) | 324,898.39 (291,764.24-361,795.42) | 7,903,725.27 (6,733,554.90-9,277,250.14) | 36,039,871.49 (16,151,560.36-80,417,761.99) | 2,583,479.74 (1,726,376.98-3,866,112.47) | 195,066.99 (179,226.91-212,307.02) | 4,289,916.47 (3,518,488.96-5,230,479.19) | 25,520,930.67 (16,307,915.97-39,938,757.55) | 2,063,976.89 (1,255,990.01-3,391,747.20) | 129,425.75 (116,154.32-144,213.53) | 3,652,291.74 (3,178,411.16-4,196,824.85) |
| **2039** | 56,058,697.82 (34,353,513.32-91,477,618.97) | 4,687,621.32 (3,109,473.41-7,066,725.03) | 339,502.02 (303,895.00-379,281.06) | 8,222,422.49 (6,929,581.18-9,756,467.20) | 38,385,712.80 (16,010,896.46-92,028,760.01) | 2,708,545.26 (1,747,592.51-4,197,899.33) | 203,802.10 (186,801.82-222,349.51) | 4,473,051.24 (3,618,945.32-5,528,734.37) | 26,506,423.98 (16,290,590.00-43,128,610.57) | 2,135,115.53 (1,243,399.12-3,666,335.52) | 135,266.00 (121,002.93-151,210.32) | 3,791,868.82 (3,269,209.27-4,398,087.72) |
| **2040** | 58,536,068.26 (34,456,302.21-99,443,964.31) | 4,874,796.55 (3,126,339.98-7,601,105.96) | 354,762.05 (316,559.11-397,575.38) | 8,553,970.34 (7,129,782.62-10,262,642.28) | 40,884,245.33 (15,839,428.11-105,529,158.32) | 2,839,665.16 (1,767,370.60-4,562,539.54) | 212,928.36 (194,709.53-232,851.90) | 4,664,003.97 (3,721,233.61-5,845,624.13) | 27,529,972.21 (16,255,928.81-46,622,950.84) | 2,208,706.10 (1,229,428.41-3,968,008.69) | 141,369.80 (126,065.49-158,532.04) | 3,936,780.03 (3,361,983.43-4,609,849.30) |
